# From Wave Energy to Electricity: Functional Design and Performance Analysis of Triboelectric Nanogenerators

**DOI:** 10.1007/s40820-025-01811-3

**Published:** 2025-06-16

**Authors:** Ying Lou, Mengfan Li, Aifang Yu, Junyi Zhai, Zhong Lin Wang

**Affiliations:** 1 Center for High-Entropy Energy and Systems, Beijing Key Laboratory of Micro-Nano Energy and Sensor, Beijing Institute of Nanoenergy and Nanosystems, , Chinese Academy of Sciences, Beijing, 101400 People’s Republic of China; 2 Center on Nanoenergy Research, Institute of Science and Technology for Carbon Peak & Neutrality; Key Laboratory of Blue Energy and Systems Integration (Guangxi University), Education Department of Guangxi Zhuang Autonomous Region; School of Physical Science & Technology, Guangxi University, Nanning, 530004 People’s Republic of China; 3 School of Nanoscience and Engineering, University of Chinese Academy of Science, Beijing, 100049 People’s Republic of China

**Keywords:** Triboelectric nanogenerator, Functional design, Blue energy, Electrical performance, Sustainability analysis

## Abstract

Triboelectric nanogenerators (TENGs) offer a self-sustaining power solution for marine regions abundant in resources but constrained by energy availability. Since their pioneering use in wave energy harvesting in 2014, nearly a decade of advancements has yielded nearly thousands of research articles in this domain. Researchers have developed various TENG device structures with diverse functionalities to facilitate their commercial deployment. Nonetheless, there is a gap in comprehensive summaries and performance evaluations of TENG structural designs. This paper delineates six innovative structural designs, focusing on enhancing internal device output and adapting to external environments: high space utilization, hybrid generator, mechanical gain, broadband response, multi-directional operation, and hybrid energy-harvesting systems. We summarize the prevailing trends in device structure design identified by the research community. Furthermore, we conduct a meticulous comparison of the electrical performance of these devices under motorized, simulated wave, and real marine conditions, while also assessing their sustainability in terms of device durability and mechanical robustness. In conclusion, the paper outlines future research avenues and discusses the obstacles encountered in the TENG field. This review aims to offer valuable perspectives for ongoing research and to advance the progress and application of TENG technology.

## Highlights

- Systematically expounding functional design of reported triboelectric nanogenerators (TENGs).
- Conducting an extensive comparison of the power conversion efficiencies of TENGs in air and water wave environments.
- Comprehensively assessing the existing challenges and delineating the future pathways for development.

## Graphical abstract

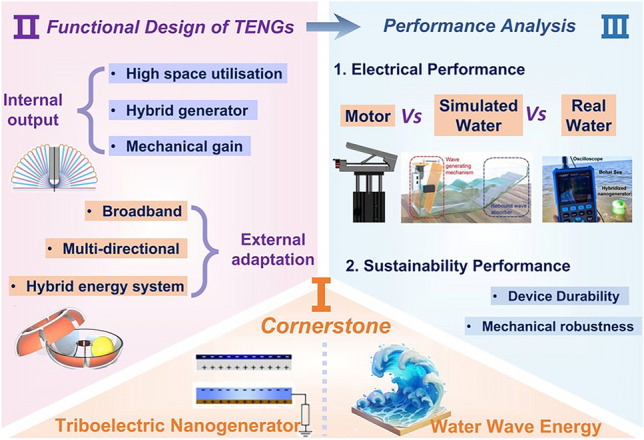

## Introduction

The global population surge has heightened the demand for traditional energy sources like oil, natural gas, and coal [1]. Since 1990, the global population has tripled, while energy consumption has increased tenfold, from 1,000 to 10,000 GW [2]. Just 10% of the world’s population consumes 90% of fossil fuel resources, which are finite and non-renewable, exacerbating resource depletion and environmental pollution. As a result, global energy policies are increasingly shifting toward renewable sources. Among these, ocean energy stands out as one of the most promising options. It encompasses various forms, including tidal, ocean current, wave, temperature gradient, and salinity gradient energy [3]. Wave energy, in particular, is available 90% of the time, compared to just 20% to 30% for solar and wind energy [4]. Its energy density can reach up to 30 kW m^−1^, which is ten times that of solar and five times that of wind energy. Efficient utilization of these renewable sources depends on both their energy density and the methods employed to harness them.

**Fig. 1:**
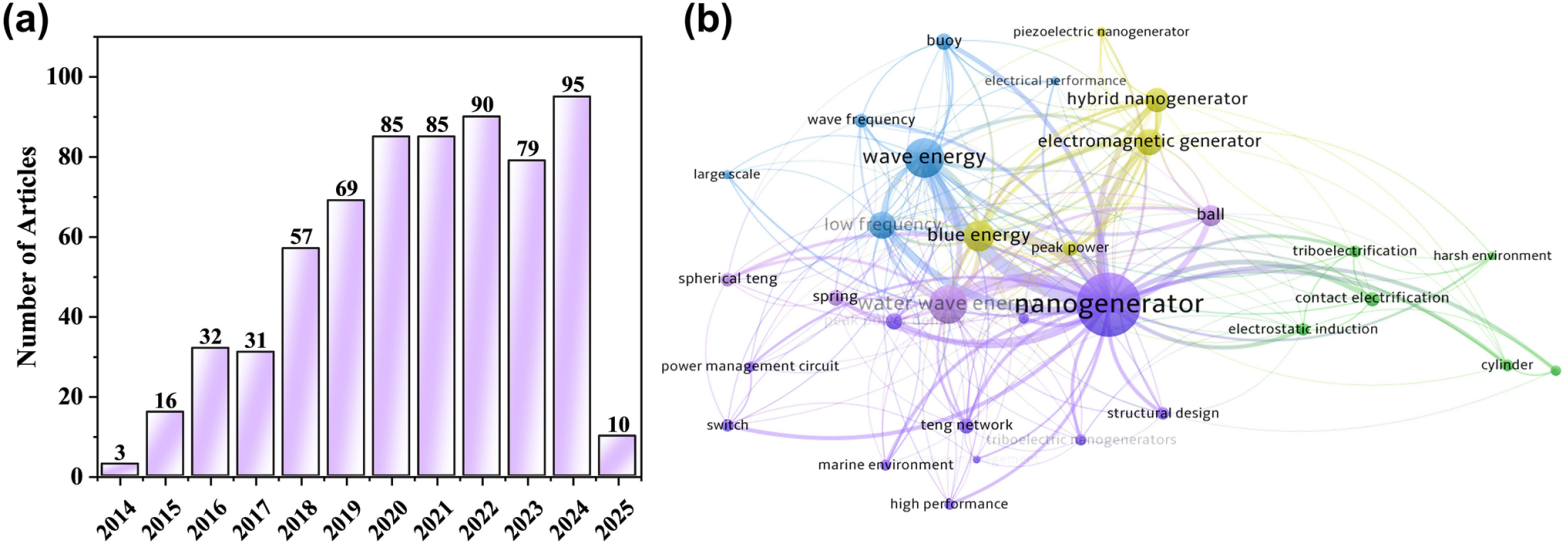
**a** A literature survey of annual publications on TENG applications in ocean energy, based on the SCI database up to February 2025. **b** Research hotspots in this field

In 2012, Zhonglin Wang introduced the triboelectric nanogenerator (TENG), which converts small mechanical energy into electrical energy via triboelectrification and electrostatic induction [5–8]. TENGs are now widely used in micro-nano-energy [9, 10], self-powered sensing [11–13], high-voltage power sources [14], and blue energy [15]. Their low mass, cost-effectiveness, and high efficiency make them ideal for harvesting low-frequency mechanical energy [16–18], such as from water waves [19], while traditional electromagnetic generators (EMGs) are better suited for high-frequency energy [3, 20]. Since its debut in water wave energy harvesting in 2014, the technology has advanced significantly [21–23]. Owing to their outstanding performance and promising potential, the annual number of publications on TENGs for water wave energy harvesting has exhibited a steady and sustained growth trend (Fig. 1a). A keyword analysis of more than 600 relevant publications (Fig. 1b) reveals prominent terms such as "nanogenerator" and "blue energy," with emerging research hotspots including "triboelectrification," "hybrid nanogenerator," "structural design," "low frequency," "power management circuit," "marine environment," and "large scale." It can be inferred that the continuous research into TENG principles, electrical output, and aquatic performance is essential for commercialization.

**Fig. 2:**
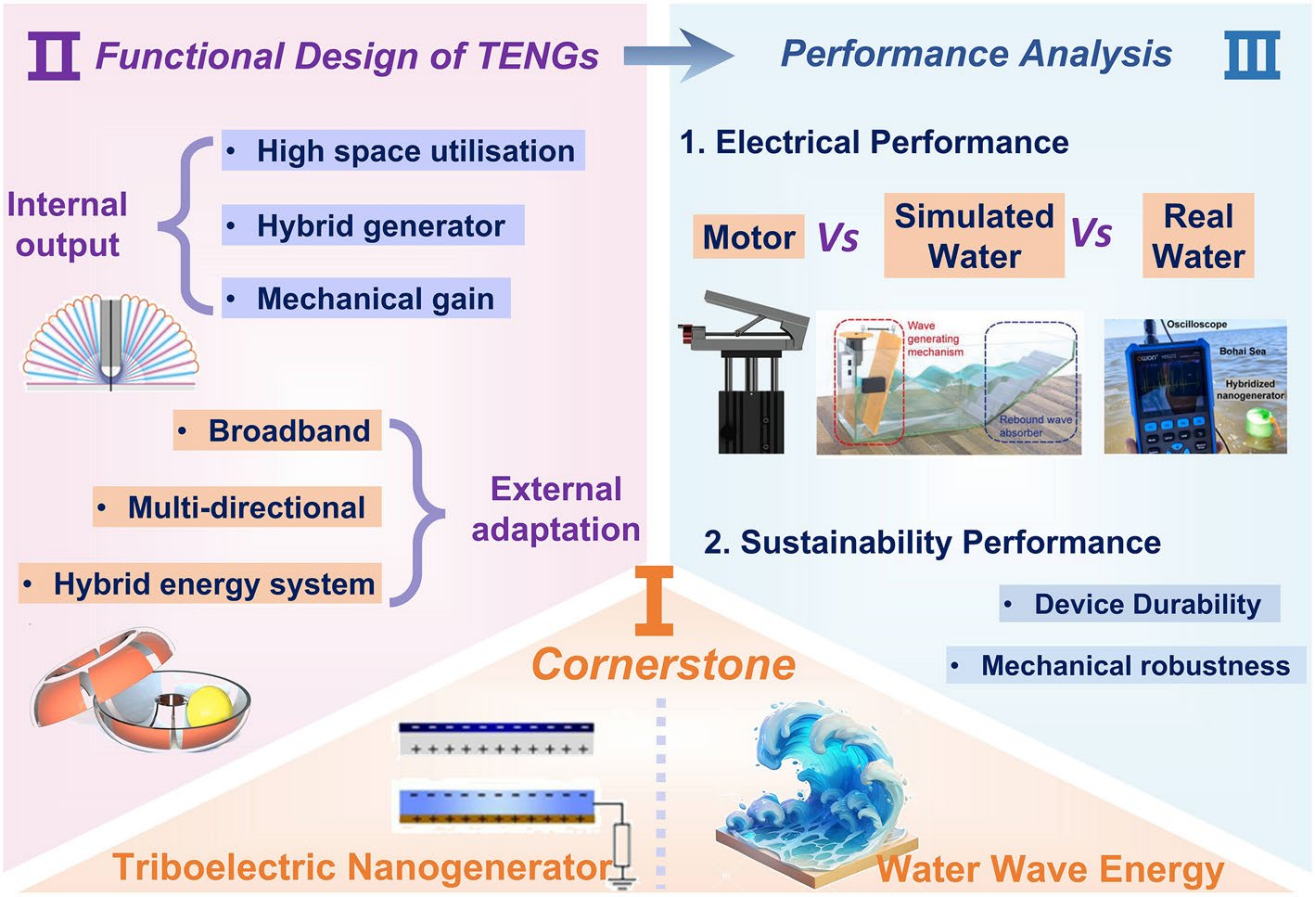
Functional design and performance analysis of TENG. Reproduced with permission [36]. Copyright 2019, The Royal Society of Chemistry. Reproduced with permission [37]. Copyright 2019, Elsevier. Reproduced with permission [38, 39]. Copyright 2024, 2019, Wiley–VCH. Reproduced with permission [40]. Copyright 2024, Elsevier

The rapid development of TENG technology has been well documented in numerous high-quality reviews covering various specialized domains [16, 24–27], and particular attention has been paid to water wave energy harvesting through comprehensive analyses of structural designs, performance optimization, and application scenarios [15, 28–35]. However, there remains a lack of systematic summaries regarding the functional design and comprehensive analysis of different structures in water environments. In light of this, this review distinguishes itself by systematically exploring six design trends of TENGs: high space utilization, hybrid generator, mechanical gain, broadband response, multi-directional operation, and hybrid energy-harvesting systems (Fig. 2). Additionally, we comprehensively evaluate the electrical performance in various aquatic environments and sustainability of devices. Finally, we discuss future research directions and challenges. We hope this review will serve as a valuable reference for both academia and industry, facilitating the commercialization of TENGs.

## Working Mode of TENG

**Fig. 3:**
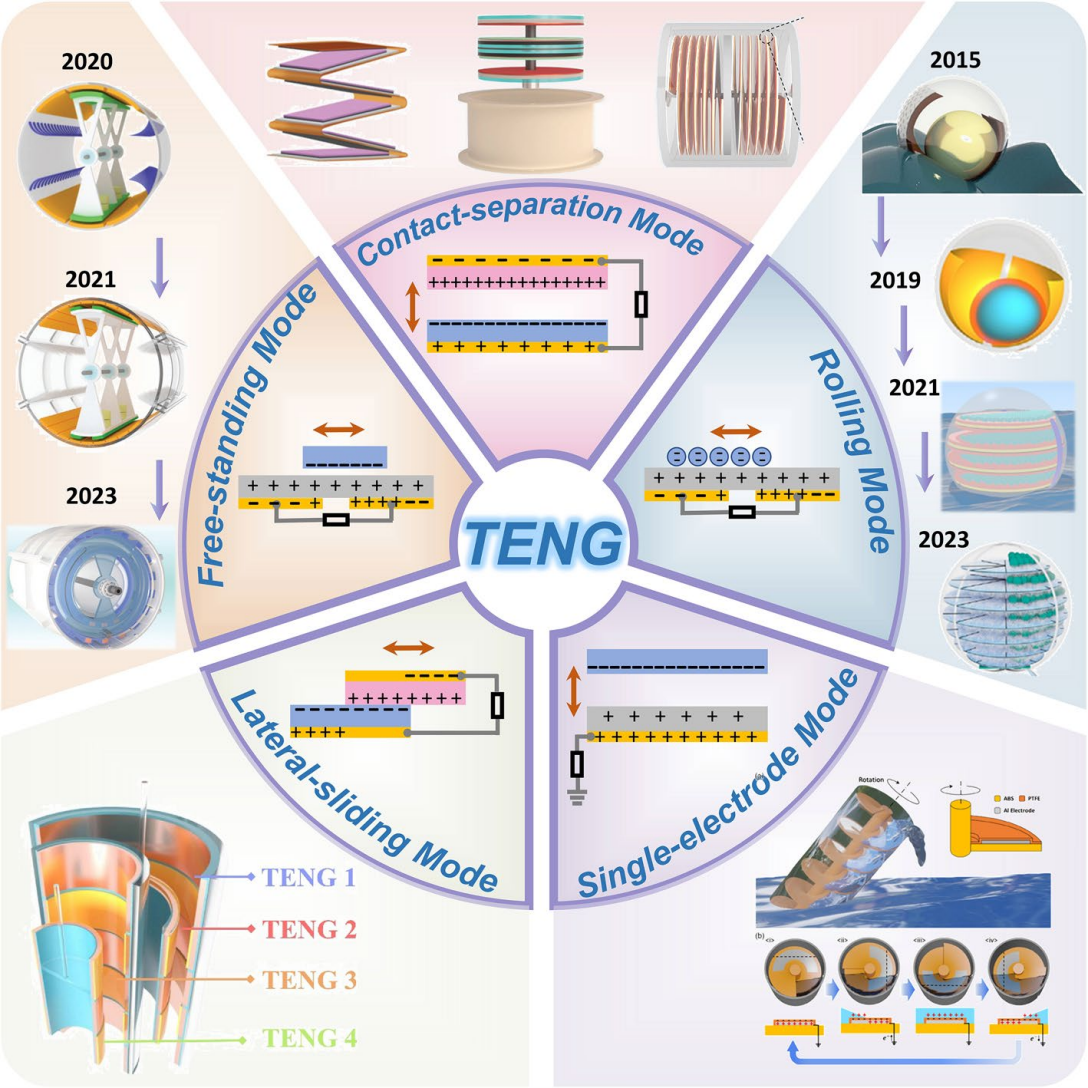
Working mode of TENG. Reproduced with permission. [44, 50] Copyright 2022, 2024, Cell Press. Reproduced with permission. [38, 43, 45–47, 51, 53] Copyright 2018, 2024, 2022, 2021, 2024, 2015, 2021, Wiley–VCH. Reproduced with permission. [48] Copyright 2019, Elsevier. Reproduced with permission. [49] Copyright 2021, American Chemical Society. Reproduced with permission. [52] Copyright 2024, Springer

TENGs operate based on Maxwell's displacement current mechanism, coupled with triboelectrification and electrostatic induction [41]. When dissimilar materials contact and separate, surface charge transfer creates a potential difference that drives electron flow. Over the past decade, the fundamental working modes of TENGs have expanded from the original four [22] to five [42]], each with unique characteristics for blue energy applications (Fig. 3).

### Contact-separation mode

This most prevalent mode generates electricity through periodic contact and separation of triboelectric materials under wave motion. While offering manufacturing simplicity and high output (particularly in stacked configurations [38, 43, 44]), its energy generation is limited by operational frequency. Typically limited to single activation per wave cycle, unlike higher-frequency modes.

### Freestanding mode

This mode employs a movable dielectric layer between stationary electrodes to generate current through electrostatic induction. The cylindrical design is widely used in blue energy applications. Through progressive optimization of materials and structures, evolving from PTFE brushes to rabbit hair brushes and ultimately to multilayer structures, researchers have significantly enhanced output efficiency while minimizing resistance [45–47]. The mode's key advantage lies in its prolonged output duration post-activation, though spatial efficiency requires improvement.

### Rolling mode

An advanced derivative of freestanding mode, this mode utilizes spherical rollers to create potential differences with minimal resistance. Its evolution from single-sphere to multilayer designs has enhanced both omnidirectional response and spatial efficiency, making it particularly suitable for compact devices [48–51]. However, However, material durability in marine environments remains a critical challenge.

### Lateral sliding mode

Despite its theoretical output advantages, few studies have applied it to blue energy [52], mainly because high sliding friction resistance hinders efficient wave energy conversion. This fundamental limitation has restricted its practical engineering implementation.

### Single-electrode mode

The single-electrode mode TENG feature a simplified design comprising a dielectric material and an electrode. In blue energy systems, water often serves as the dielectric [53], leveraging its adaptive properties to achieve effects unattainable with solid dielectrics. However, lower charge transfer and potential differences result in limited energy output.

## Functional Design of Advanced TENGs

Water plays a dual critical role in TENGs for ocean energy harvesting, serving as both an adaptive triboelectric material and a sustainable power source. While solid–liquid contact TENGs overcome the limitations of random water waves, solid–solid contact configurations show greater commercial potential for practical marine power generation. This review focuses specifically on solid–solid contact TENG designs for marine applications. Given the ocean's complex and dynamic environment, purpose-optimized device architectures are increasingly needed to maximize energy conversion efficiency. Over the past decade, TENG designs have evolved toward higher power output and improved environmental adaptability. We systematically examine key design considerations including high space utilization, hybrid generator, mechanical gain, broadband response, multi-directionality, and hybrid energy-harvesting systems.

### High Space Utilization Design

Multilayer design is a pivotal method for enhancing space efficiency in TENG devices. By stacking multiple units in a limited space, it maximizes the power-generating area per unit volume. Under periodic wave excitation, layered generator units move nearly synchronously. When connected in parallel, they increase short-circuit current and output power. Among the five working modes of TENGs [42], over 90% of solid–solid contact devices employ the contact-separation, rolling, and freestanding modes, with multilayer designs being especially common in these three modes.

#### Contact-Separation Mode TENG

**Fig. 4:**
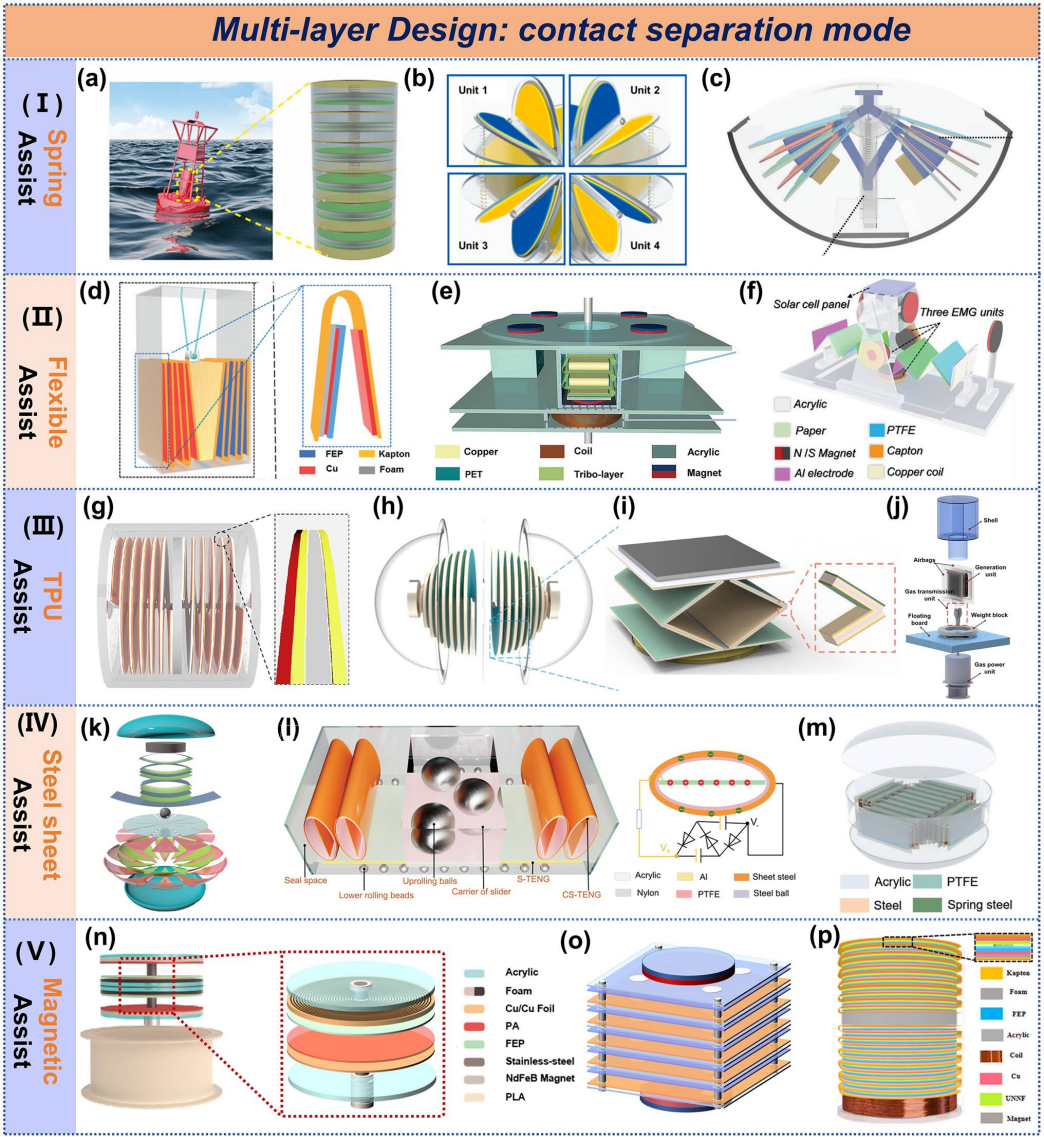
Multilayer design of contact-separation mode TENG. **a**, **b** Self-powered intelligent buoy system, a spring-assisted structure. Reproduced with permission [54, 56]. Copyright 2019, 2021, Elsevier. **c-f** Butterfly-inspired TENG, bifilar-pendulum-assisted multilayer-structured TENG, space confined multilayer-stack TENG, nonencapsulative pendulum-like paper–based hybrid nanogenerator. Reproduced with permission [55, 57–59]. Copyright 2019, 2021, 2023, 2019, Wiley–VCH. **g** Anaconda-shaped spiral multi-layered TENG. Reproduced with permission [44]. Copyright 2022, Elsevier. **h** Multi-layered helical spherical TENG. Reproduced with permission [60]. Copyright 2023, Wiley–VCH. **i** Elastic self-recovering hybrid nanogenerator. Reproduced with permission [61]. Copyright 2024, MDPI. **j** Gas-assisted TENG. Reproduced with permission [62]. Copyright 2024, Elsevier. **k-n** Oblate spheroidal TENG, versatile blue energy TENG, 0.5 m TENG, high-coupled magnetic-levitation hybrid nanogenerator. Reproduced with permission [38, 63–65]. Copyright 2019, 2023, 2022, 2024, Wiley–VCH. **o** A multilayer magnetic suspension hybrid nanogenerator. Reproduced with permission [66]. Copyright 2025, The Royal Society of Chemistry. **p** a magnetic suspension damped hybrid nanogenerator. Reproduced with permission [67]. Copyright 2025, Wiley–VCH

The contact-separation mode is the most common mode in multilayer design structures. Depending on the realization, they can be classified as spring-assisted, flexible substrate, TPU-assisted, steel sheet-assisted, and magnetic-assisted. A self-powered intelligent buoy system was fabricated using spring-assisted techniques (Fig. 4a), with an average output power density of 13.2 mW m^−2^ in its multi-layered power-generating units, and achieved sustained wireless transmission [54]. A symmetrical butterfly-inspired TENG was fabricated, which can synchronize to contact and separate via an intermediate spring linkage (Fig. 4c) [55]. Liang et al. successfully achieved the motion of four sets of TENG units in a sphere using spring assistance (Fig. 4b), combined with charge excitation circuits to manage the output power up to 23.3 mA of short-circuit current and 16.6 mW of power [56], which is not limited by the direction of the water waves in this structure compared to Lei's design [55].

Kapton [57], PET [58], and origami [59] are commonly employed in flexible substrate design (Fig. 4d-4f), with Kapton being the most widely used. A spherical TENG structure was constructed using a Kapton substrate with spring assistance, achieving successful wireless transmission [68]. The research team further optimized circuit management in 2020, increasing the current to 25.1 mA [69]. That same year, they integrated the structure for multi-directional functionality to address complex marine environments [70]. Other research teams have also adopted Kapton, some without spring assistance [71–73]. Zhang et al. achieved a power density of 200 W m⁻^3^ through wedge-shaped oscillation for unit compression [57], and subsequently introduced EMG and PENG to reach a power density of 358 W m⁻^3^, demonstrating the effective spatial utilization of multilayer designs [74].

Due to its suitability for 3D printing, TPU is widely used. Anaconda TPU was used to create a symmetrical multilayer TENG structure (Fig. 4g), achieving a spatial efficiency of 93.75% and a peak power density of 347 W m^−3^ [44]. Liu [60], Wang [61], and Gao [62] explored various TPU framework shapes to enhance electrical output (Fig. 4h-4j). Spring steel sheets were preferred as substrates due to their excellent elasticity and rebound properties, making them ideal for the specific designs shown in Fig. 4k and 4l [63, 64]. Feng et al. were the first to fabricate a 0.5 m TENG using spring steel sheets (Fig. 4m), achieving an exceptionally high charge transfer of 67.2 μC [65].

Although multilayer designs enhance spatial utilization and power density, spring are limited by length and stress, making extensive layering challenging. Kapton, PET, origami, and TPU have limited lifespans and are unsuitable for harsh marine environments. Steel sheets are complex to prepare and prone to corrosion, restricting their broader application. Magnetic-assisted technology presents a promising alternative. Li et al. developed a magnetic-levitation structure based on like-pole repulsion (Fig. 4n), which not only simplifies the design but also ensures long-term stability due to permanent magnet properties. Notably, The magnetic repulsion force enables rapid separation of triboelectric layers, achieving a remarkable short-circuit current of 146 μA from a compact 90 mm diameter planar structure [38]. Further optimization led to a multilayer magnetic suspension hybrid nanogenerator with improved space utilization (Fig. 4o), delivering an ultra-high output current of 45 mA and a peak power density of 631 W m^−3^ [66]. Similarly, Zhang’s team introduced a magnetic suspension damped hybrid nanogenerator with a record power density of 628.9 W m^−3^ (Fig. 4p), where the TENG unit alone contributed 1.43 mA short-circuit current and 98.8% space utilization [67]. Lou et al. further advanced integration by leveraging "like-pole repulsion and opposite-pole attraction" to couple three generation units, achieving 91.9% system space utilization [75].

These studies demonstrate that while multilayer configurations enhance spatial efficiency and power output, their optimization requires careful consideration of material durability, environmental resilience, and engineering feasibility.

#### Rolling Mode TENG

The rolling mode has recently become a hotspot in multilayer design research due to its good adaptability to variations in wave frequency and amplitude. TENGs operating in this mode can continuously harvest energy throughout the entire motion cycle by utilizing the continuous movement of water waves, enabling higher energy conversion efficiency. The following review will cover advancements in arc rolling, flat rolling, grid rolling, and different housing structure design based on current literature.

**Fig. 5:**
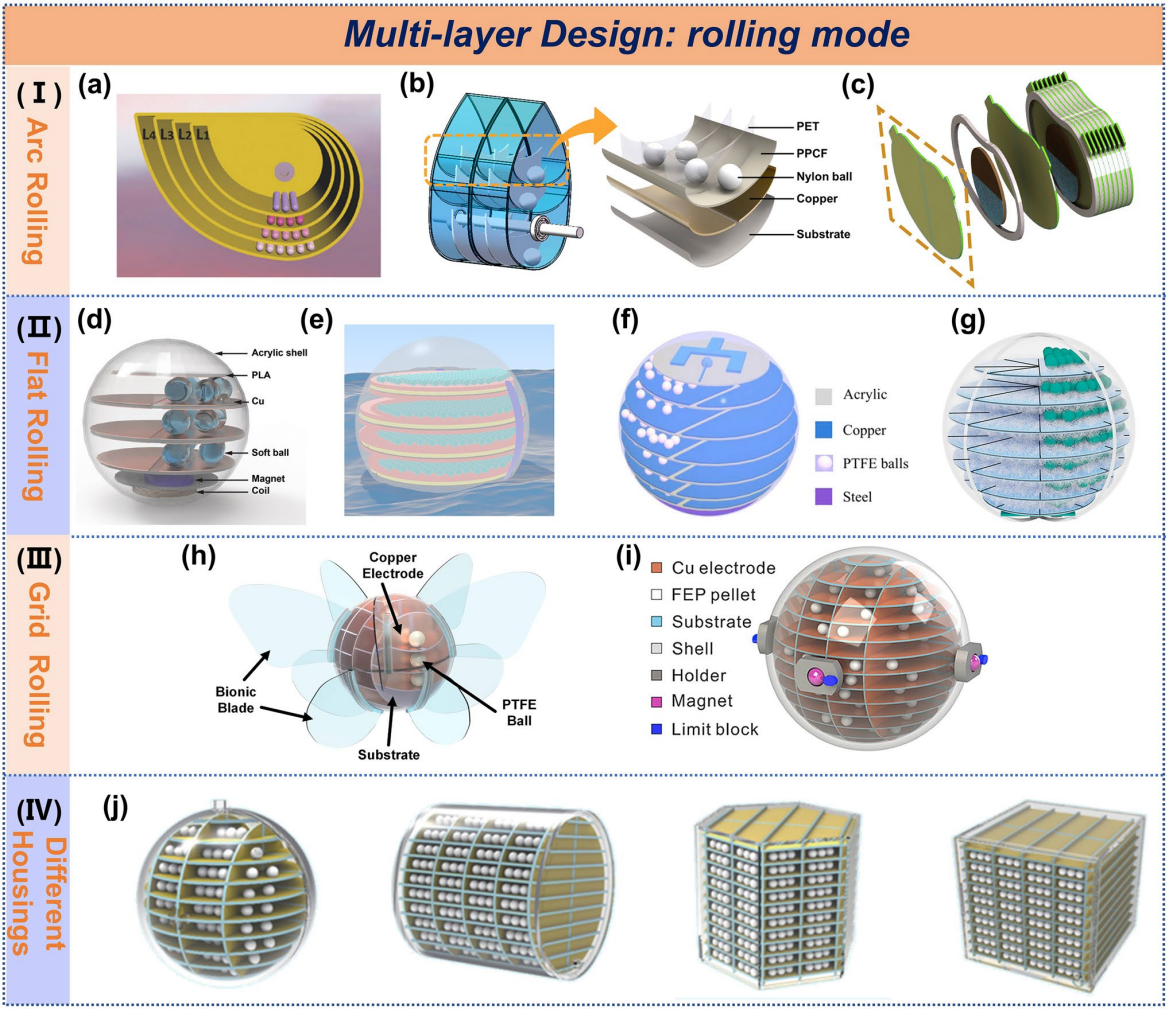
Multilayer design of rolling mode TENG. **a** Duck-shaped TENG. Reproduced with permission. [76] Copyright 2017, Wiley–VCH. **b** Anodding duck structure multi-track freestanding triboelectric-layer nanogenerator. Reproduced with permission [78]. Copyright 2021, American Chemical Society. **c** Stacked pendulum-structured TENG. Reproduced with permission [79]. Copyright 2019, Elsevier. **d** Soft ball-based TENG. Reproduced with permission [80]. Copyright 2023, Wiley–VCH. **e** Spherical TENG. Reproduced with permission [49]. Copyright 2021, American Chemical Society. **f-i** Spherical TENG, spherical TENG featuring multilayer ‘‘sliced-pizza-shaped’’ electrodes, bioinspired butterfly wings TENG, macroscopic self-assembly network of encapsulated TENG. Reproduced with permission [50, 81–83]. Copyright 2023, 2024, 2022, 2019, Elsevier. **j** Four structures sphere TENG. Reproduced with permission [84]. Copyright 2023, Springer

In the arc rolling design, the duck-shaped structure is the most common. As shown in Fig. 5a, a fully enclosed duck-shaped TENG was developed with an internal energy-harvesting unit, which was extended from a single layer to four layers to enhance current output [76]. A theoretical analysis of this structure was also conducted [77], while improvements to the shell structure and tracks resulted in a peak power density of 4 W m^−3^ (Fig. 5b) [78]. Inspired by pendulum designs, a highly stacked TENG use area contact instead of point contact for each generating unit, successfully achieving a peak power density of 14.71 W m^−3^ (Fig. 5c) [79]. However, the irregular shape of the duck-shaped structure limits device size and center of mass. Consequently, researchers began focusing on flat rolling structures.

Building on prior work [48], soft spheres were designed to increase the contact area between the friction layer and electrodes (Fig. 5d), extending to three layers with an electrical output of 0.5 mW [80]. Further increases in the density of small spheres and layer count to five resulted in a peak power density of 20.57 W m^−3^, as depicted in Fig. 5e [49]. Extending to six layers, a peak power density of 21.3 W m^−3^ was achieved (Fig. 5f), along with the integration of an energy management circuit for hydrogen production [81]. Hong et al. increased the layers to seven and used pizza-shaped electrodes (Fig. 5g), achieving a peak power density of 13 W m^−3^ at an ultra-low frequency of 0.6 Hz [50].

Researchers aspire to obtain higher electrical output by continuously accumulating the number of layers of power generation units. It is more desirable to achieve the collection of omnidirectional water waves to improve the energy capture efficiency. Similar to the orbital design of the nodding duck-like structure, networked compartments have been developed to achieve high output and capture waves from various directions. A butterfly-wing-inspired TENG, as shown in Fig. 5h, has been developed to respond sensitively to water waves [82], while a three-dimensional electrode structure in Fig. 5i achieves an average power density of 8.69 W m^−3^ [83]. In addition to the unit design, the shape of the housing plays a crucial role in performance. Various shapes, including spherical, cylindrical, regular hexagonal, and cubic, have been explored based on three-dimensional electrodes (Fig. 5j), with optimization leading to an average power density of 10.08 W m^−3^ for the cubic shape [84]. These findings highlight the promising commercial potential of multilayer designs.

#### Freestanding Mode TENG

**Fig. 6:**
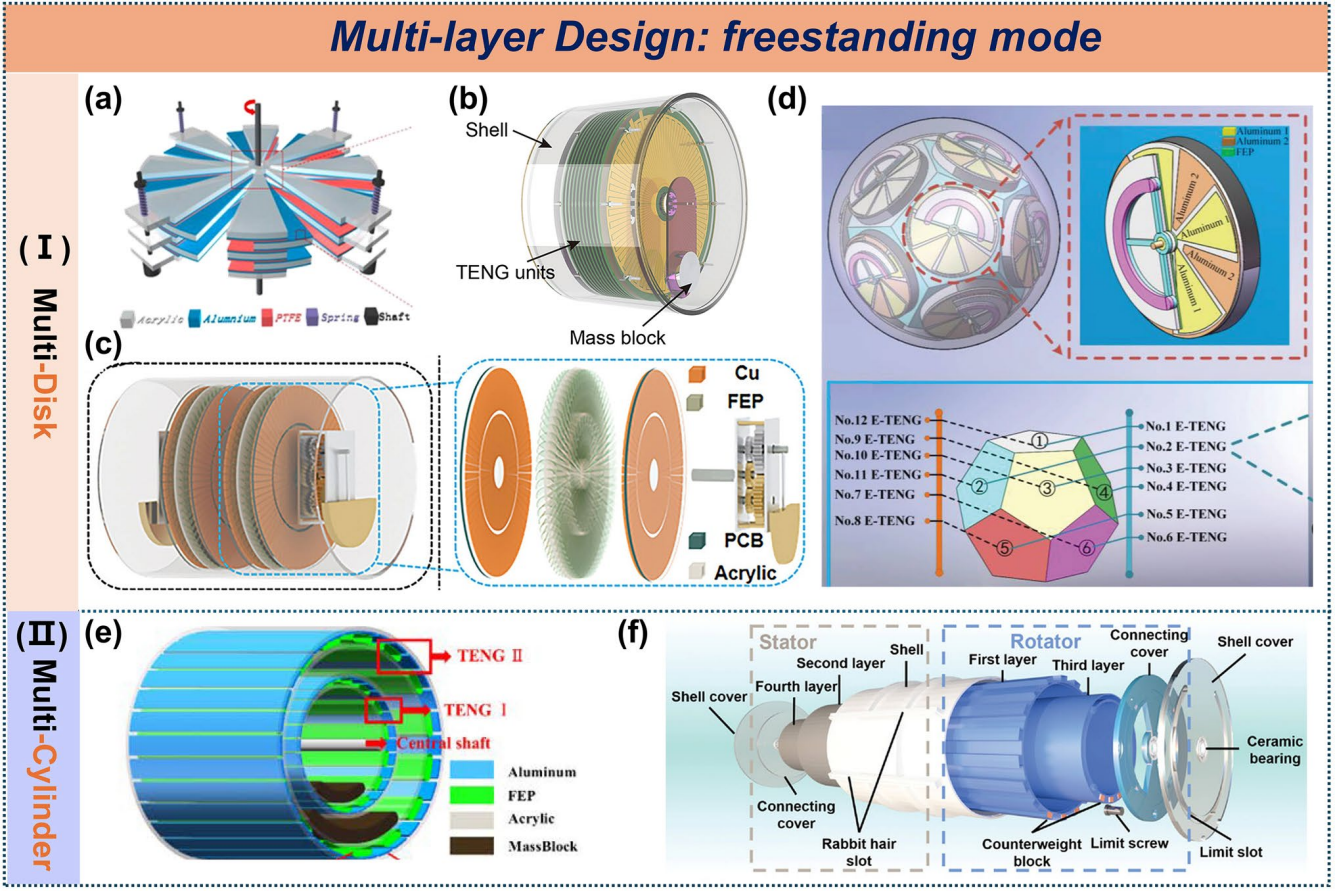
Multilayer design of freestanding mode TENG. **a** multi-layered disk TENG, **b** high-performance tandem disk TENG. Reproduced with permission [21, 85]. Copyright 2014, 20,119, Elsevier. **c** Self-adaptive rotating TENG and **d** spherical eccentric structured TENG. Reproduced with permission [86, 87]. Copyright 2023, 2022, Wiley–VCH. **e** Multi-cylinder-based TENG. Reproduced with permission [88]. Copyright 2023, MDPI. **f** Multi-layered swing-structured TENG. Reproduced with permission [47]. Copyright 2024, Wiley–VCH

In multilayer designs, multilayer disks and multilayer cylindrical structures represent the primary types of freestanding TENGs. Xie et al. first applied multilayer disk structures to water energy harvesting (Fig. 6a), achieving a peak power density of 2.68 kW m^−3^ [21]. A multilayer radial grating disk with pendulum assistance has also been designed to convert low-frequency water waves into high-frequency electrical signals (Fig. 6b), reaching an average power density of 7.3 W m^−3^. The integration of a transformer boosted the output current by 17-fold, enabling real-time water quality monitoring [85]. Zhang et al. introduced a gear structure that doubled the kinetic energy utilization (Fig. 6c), achieving an average mass power density of 45.18 mW/kg, ten times that of comparable EMGs [86]. Contrastingly, Qu et al. arranged disks in an icosahedral configuration to optimize wave capture from various directions (Fig. 6d), successfully powering a thermometer [87].

Cylindrical structures, favored for their sustained movement under single wave triggers, still face space utilization challenges. To enhance space efficiency, dual-layer TENG units were developed (Fig. 6e), achieving a peak power density of 31.23 W m^−13^ across a 2.25–4 Hz bandwidth [88]. Further improvements in space utilization were made with the design of a four-layer swinging TENG (Fig. 6f), which attained a peak power density of 15.18 W m^−3^ Hz⁻^1^ under 0.8 Hz water waves [47].

### Hybrid Generators Design

Hybrid generator systems, which often integrate TENGs, EMGs, piezoelectric generators (PENGs), and solar cells, have emerged as an effective strategy for efficient and stable water wave energy conversion [89]. The working principle of these hybrid systems relies on frequency-band complementarity. TENGs effectively harvest low-frequency (0.1–2 Hz) wave energy through relative motion. EMGs convert high-frequency (> 5 Hz) mechanical vibrations into electrical energy via the cutting motion of coils in magnetic fields. PENGs capture intermediate- frequency (2–5 Hz) energy through piezoelectric deformation. Together, they form a full-band energy-harvesting network. Solar cells serve as a supplementary power source under illumination conditions to compensate for the intermittency of ocean waves. This synergistic integration not only enhances energy conversion efficiency but also improves the stability and reliability of the system.

**Fig. 7:**
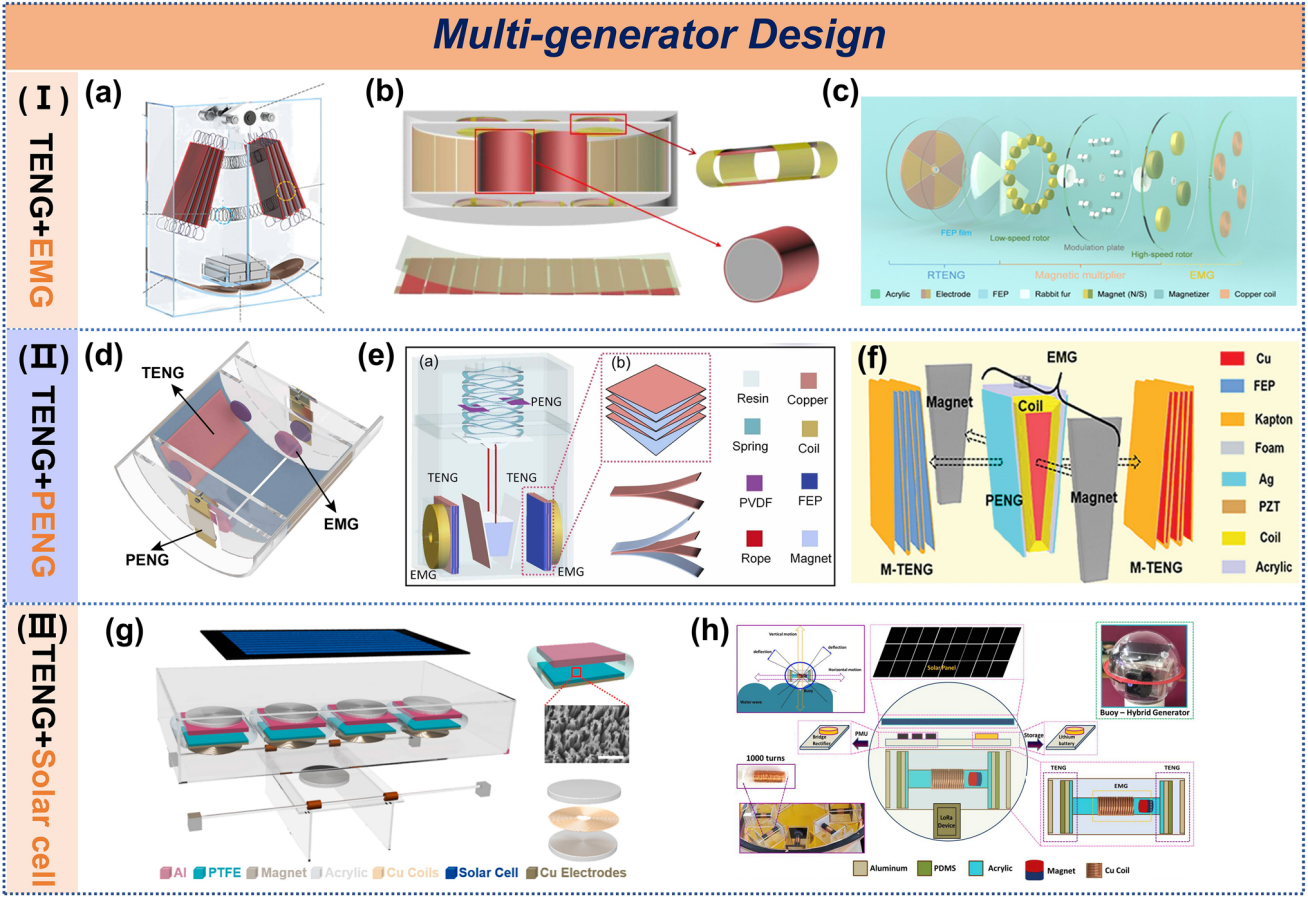
The multi-generators design of TENG. **a** Hybrid wave energy-harvesting nanogenerator. Reproduced with permission [90]. Copyright 2021, Wiley–VCH. **b** Fully-packaged ship-shaped hybrid nanogenerator. Reproduced with permission [93]. Copyright 2019, Elsevier. **c** Magnetic-multiplier-enabled hybrid generator. Reproduced with permission [94]. Copyright 2023, American Association for the Advancement of Science. **d** Triboelectric-electromagnetic-piezoelectric hybrid energy harvester, **e** spring pendulum coupled hybrid energy harvester. Reproduced with permission [40, 95]. Copyright 2022, 2024, Elsevier. **f** Bifilar-pendulum coupled hybrid nanogenerator. Reproduced with permission [74]. Copyright 2022, Wiley–VCH. **g** Multifunctional hybrid power unit, **h** fully packed spheroidal hybrid generator. Reproduced with permission [96, 97]. Copyright 2017, 2020, Wiley–VCH

The integration of TENG with EMG is currently one of the most prevalent hybrid approaches. EMG utilize permanent magnets to establish a stable magnetic field and generate induced electromotive force through electromagnetic induction. In some hybrid generators, magnets serve not only to provide the magnetic field but also to facilitate weighting, triggering, and mechanical regulation. For instance, a multilayer TENG was designed with a central pendulum featuring a symmetrical axis (Fig. 7a), where magnetic blocks enhance the pendulum's inertial motion characteristics [90]. In another approach, magnetic blocks were used to allow the pendulum to swing freely in any direction, while the bottom electrodes were shaped in a Tai Chi pattern to improve the wireless transmission efficiency of the triboelectric-electromagnetic hybrid generator [91]. A similar design incorporated magnets to trigger the mechanism, where rolling magnetic balls facilitated the TENG's back-and-forth sliding motion and effectively cut magnetic field lines [92]. In contrast, a boat-shaped hybrid generator alternates between magnetic attraction and repulsion during rolling (Fig. 7b), enabling the TENG to contact and separate while simultaneously driving the EMG in rolling mode [93]. Additionally, optimizing the placement of magnets within a disk allowed for magnetic acceleration, while frequency division was employed to operate the EMG at higher frequencies (Fig. 7c), addressing the typically lower efficiency of EMGs compared to TENGs [94].

The integration of PENG and solar cell with TENG represents a promising approach for maximizing energy utilization. A hybrid system combining TENG, PENG, and EMG was fabricated by introducing a cantilever beam structure (Fig. 7d), which enhanced space utilization and achieving a power density of 5.73 W m^−3^ [95]. Zhang et al. [74] and Gao et al. [40] utilized a wedge-shaped pivot mechanism to enable the operation of multilayer contact-separation TENGs on both sides (Fig. 7e, f). The placement of PENG varied slightly, and for EMGs, the former employed a transverse magnetic flux cutting mode with more significant flux variation, while the latter used a longitudinal cutting mode, resulting in slightly lower electrical output. Shao et al. [96] and Chandrasekhar et al. [97] incorporated solar cells into the top of the triboelectric-electromagnetic hybrid structure to provide power support under calm wave conditions (Fig. 7g, h).

For a more detailed comparison of hybrid generators, refer to Table 1. Comprehensive analysis reveals that magnets should serve beyond mere magnetic field provision. In particular, magnetic components should be able to achieve mechanical regulation to create a synergistic effect where 1 + 1 > 2, which represents a primary objective in hybrid structures. Moreover, the stark imbalance in unit power contributions—with reported ratios varying by hundreds or thousands of times—poses a critical challenge. Addressing these aspects will steer future research on hybrid generator architectures.

**Table 1:** The comparison table of design and electrical performance for hybrid nanogenerators

Device structure	Hybrid Module	Mode	Magnet Shape	Magnet's role	Excitation	Power /Power Density	Power Ratio	Refs
Rolling	TENG	Rolling	Square	Magnetic field	2 Hz	1.05 μW/cm^3^	1:1.25	[98]
	EMG	Transverse cutting				1.32 μW/cm^3^		
Rolling	TENG	Contact-separate	Sphere	Magnetic field	1.0 Hz	79 W m^–3^	/	[99]
	EMG	Transverse cutting				0.76 mW		
Sliding	TENG	Contact-separate	Cylinder	1. Trigger	2 Hz	31.5 μW	2.12:1	[96]
	EMG	1.Transverse cutting		2. Magnetic field		66.9 μW		
		2.Longitudinal cutting						
	Solar cell	Photovoltaic effect			T:100%	0.14 mW cm^−2^	/	
Cylinder	TENG	Contact-separate	Cylinder	1. Trigger	100 rpm	90.7 μW	1.14:1	[100]
	EMG	1.Transverse cutting		2. Magnetic field		79.6 μW		
		2.Longitudinal cutting						
Waterwheel	TENG	Rolling	Rod	1. Friction layer	0.5 Hz	67.05 mW m^−2^	/	[101]
	EMG	Transverse cutting		2. Magnetic field	1.05 Hz	3 mW		
Waterwheel	TENG-1	Single-electrode	Cylinder	Magnetic field	200 rpm	4.75 μW	1:2086	[102]
	TENG-2	Freestanding						
	EMG	Transverse cutting				9.91 mW		
Waterwheel	TENG-1	Freestanding	Cylinder	Magnetic field	300 rpm	68	2.1: 1	[103]
	EMG-1	Transverse cutting				7		
	TENG-2	Freestanding				10		
	EMG-2	Transverse cutting				30		
Absorber	TENG	Lateral sliding	Cylinder	Magnetic field	5 Hz	120 W m^−3^	1:1.8	[104]
	EMG	Transverse cutting				220 W m^−3^		
Piston	TENG	Contact-separate	Strip	Magnetic field	300 rpm	0.302mW	10:1	[105]
	EMG	Transverse cutting				0.0295 mW		
Sphere	TENG	Freestanding	1.Sphere	1. Trigger				[92]
	EMG	Transverse cutting	2.Cylinder	2. Magnetic field				
Topology	TENG	Contact-separate	Cylinder	Magnetic field	1 Hz	0.5 mW	1:8	[106]
	EMG	Longitudinal cutting				4 mW		
Paper	TENG	Contact-separate	Cylinder	1. Trigger	1 Hz	22.5 mW	16:1	[59]
	EMG	Transverse cutting		2. Magnetic field		1.39 mW		
Spring	TENG	Contact-separate	Strip	1.Counterweight	1.5 Hz	1.72 mW	1.16:1	[90]
	EMG	Transverse cutting		2. Magnetic field		1.48 mW		
Ship	TENG-1	Contact-separate	Cylinder	1. Trigger	2 Hz	800 μW	1: 9	[93]
	TENG-2	Rolling		2. Magnetic field		165 μW		
	EMG	1.Transverse cutting				9 mW		
		2.Longitudinal cutting						
Box	TENG	Rolling	Sphere	1. Trigger	1.8 Hz	0.08 mW	1: 186	[107]
	EMG	Transverse cutting		2. Magnetic field		14.9 mW		
Pendulum	TENG	Freestanding	Sphere	1.Counterweight	2.5 Hz	15.21 μW	1: 81	[108]
	EMG	Transverse cutting		2. Magnetic field		1.23 mW		
Pendulum	TENG-1	Rolling	Cylinder	1.Counterweight	1 Hz	95.4 mW	/	[109]
	TENG-2	Freestanding		2. Magnetic field				
	EMG	Transverse cutting						
Sphere	TENG	Contact-separate	Cylinder	Magnetic field	1.5 Hz	700 μW	0.35:3:40	[97]
	EMG	Transverse cutting				6 mW		
	Solar cell	Photovoltaic effect				80 mW		
Layered	TENG	Freestanding	Cylinder	1. Mechanical regulation	2.67 Hz	4.107 mW	1:2.1	[94]
	EMG	Transverse cutting		2. Magnetic field		8.688 mW		
Pendulum	TENG	Freestanding	Strip	1.Counterweight	0.1 Hz	2.71 W·m^−3^	1:2.7	[110]
	EMG	Transverse cutting		2. Magnetic field		7.45 W·m^−3^		
Swing magnetic	TENG	Freestanding	Cylinder	1.Counterweight	300 rpm	0.26 mW	1:24	[91]
	EMG	Transverse cutting		2. Magnetic field		6.2 mW		
Seesaw	TENG-1	Contact-separate	Cylinder	Magnetic field	0.7 Hz	17 W m^−3^	2.2:1	[111]
	TENG-2	Rolling				4.8 W m^−3^		
	EMG	Transverse cutting				9.8 W m^−3^		
Pendulum	TENG	Contact-separate	Trapezoidal	Magnetic field	5 m s^−2^	15 mW	15:7:2	[74]
	PENG	Piezoelectric effect				7 mW		
	EMG	Transverse cutting				2 mW		
Cantilever beam	TENG	Freestanding	Cylinder	1. Mechanical regulation	0.5 m s^−2^	3.77 mW	9:1:12	[95]
	PENG	Piezoelectric effect		2. Magnetic field		0.4 mW		
	EMG	Transverse cutting				4.87 mW		
Pendulum	TENG	Freestanding	Cuboidal	Magnetic field	5 Hz	0.18 mW	1:48	[112]
	EMG	Transverse cutting				8.6 mW		
Swing	TENG	Rolling	Cylinder	Magnetic field	0.96 m/s	0.064 mW	1:6.3	[113]
	EMG	Transverse cutting				0.403 mW		
Eccentric	TENG	Freestanding	Cylinder	1.Counterweight	1.25 Hz	0.15 mW kg^−1^	1:74	[114]
	EMG	Transverse cutting		2. Magnetic field		11.1 mW kg^−1^		
Seesaw	TENG	Rolling	Cuboidal	1. Mechanical regulation	1 Hz	161.0 μW	1:390	[115]
	EMG-1	Transverse cutting		2. Magnetic field		24.7 mW		
	EMG-2	Transverse cutting				22.5 mW		
Waterwheel	TENG-1	Freestanding	Cylinder	Magnetic field	90 rpm	1.44 mW	1:10	[116]
	TENG-2	Single-electrode				0.15 mW		
	EMG	Transverse cutting				15.9 mW		
Flapping wing	TENG	Freestanding	Cylinder	Magnetic field	0.64 m/s	37.28 mW	4:1	[117]
	EMG	Transverse cutting				9.36 mW		
Pendulum	TENG	Contact-separate	Trapezoidal	1.Counterweight	6 Hz	59.9 μW	58:1:342	[40]
	PENG	Piezoelectric effect		2. Magnetic field		1.03 μW		
	EMG	Longitudinal cutting				352 μW		
Pendulum	TENG-1	Contact-separate	Cylinder	1. Trigger	2.6 Hz	16 mW	100:1	[75]
	TENG-2	Rolling		2. Magnetic field		55 μW		
	EMG	Longitudinal cutting				0.16 mW		
Waterwheel	TENG	Freestanding	Cylinder	Magnetic field	200 rpm	3 mW	1:1.5	[118]
	EMG	Transverse cutting				4.5 mW		
Box	TENG	Single-electrode	Rod	1.Counterweight	2 Hz	85.3 μW	1:1.12	[119]
	EMG	Transverse cutting		2. Magnetic field		95.6 μW		
Turbine	TENG	Freestanding	Cylinder	Magnetic field	1 Hz	32.55 W m^−3^	1:10	[120]
	EMG	Transverse cutting				329.78 W m^−3^		
Magnetic Levitation	TENG	Contact-separate	Annular	1. Mechanical regulation	8 m s^−2^	12.17 mW	26:1	[38]
				2. Magnetic field				
	EMG	Longitudinal cutting				0.47 mW		
Shuttle	TENG	Contact-separate	Cuboidal	Magnetic field	5 m s^−2^	9.0 mW	1:3:3.8	[121]
	PENG	Piezoelectric effect				26.2 mW		
	EMG	Transverse cutting				34.2 mW		
Dragonfly	TENG	Freestanding	Cylinder	Magnetic field	0.21 m/s	4.19 mW	1:2	[122]
	EMG	Transverse cutting				8.01 mW		

### Mechanical Gain Design

To tackle the power intermittency of TENGs due to the low-frequency and random nature of water waves, precise mechanical regulation of TENG structures has been devised. Leveraging frequency modulation, inertial components within the mechanical gain structure transform the original low-frequency, erratic wave motions into high-frequency, consistent mechanical energy upon wave activation. This mechanical gain strategy not only overcomes the energy conversion efficiency bottleneck of TENGs under low-frequency excitation but also substantially improves device adaptability across various wave conditions. The main mechanical gain methods encompass spring-driven, pendulum-driven, gear-driven, and magnetic-driven mechanisms.

**Fig. 8:**
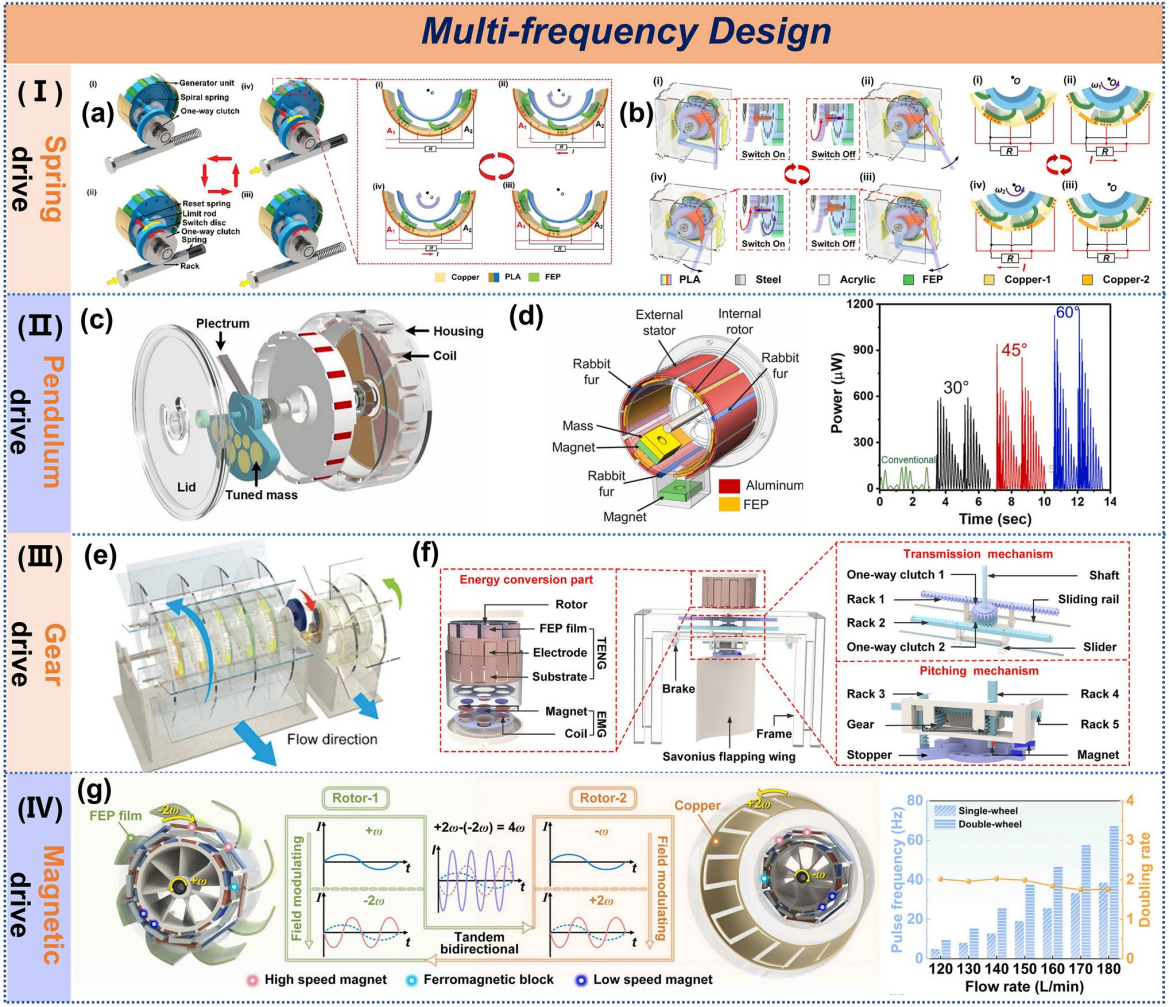
Multi-frequency design of TENG. **a** Mechanical regulation TENG. Reproduced with permission [125]. Copyright 2020, Wiley–VCH. **b** Double rocker TENG, **c** multi-purpose triboelectric-electromagnetic hybrid nanogenerator, **d** frequency-multiplied cylindrical TENG. Reproduced with permission [112, 126, 127]. Copyright 2021, 2022, 2022, Elsevier. **e** A fur-brush TENG. Reproduced with permission [128]. Copyright 2021, Wiley–VCH. **F S**avonius flapping wing triboelectric–electromagnetic hybrid generator, **g** accelerated charge transfer TENG. Reproduced with permission [117, 129]. Copyright 2024, 2024, Elsevier

Spring energy storage converts mechanical energy into elastic potential energy through the elastic deformation of springs, enabling periodic release and transforming low-frequency motion into high-frequency oscillations [123, 124]. A switch was employed to control the compression and relaxation of a helical spring, which, in conjunction with a flywheel, converted stochastic energy into stable electrical power (Fig. 8a), achieving a peak power of 2.52 mW [125]. In contrast, a double rocking arm mechanism was utilized to harvest energy (Fig. 8b), achieving a power output of 11 mW [126].

Besides spring-based systems, pendulums are also effective for multi-frequency output, converting a single trigger into sustained motion lasting seconds to minutes. A triboelectric-electromagnetic hybrid nanogenerator was designed with a spring pendulum to convert low-frequency motion into high-speed rotation (Fig. 8c), effectively triggering waves below 5 Hz [112]. To address the efficiency issues of cylindrical TENGs at low frequencies and amplitudes, a pendulum-driven system with magnets for energy storage and release was implemented (Fig. 8d), achieving a peak power density of 6.67 W m^−3^. Compared to traditional cylindrical TENGs, the mechanically adjusted system improved power output by a factor of five, reaching 0.178 mW [127].

Additionally, gear systems are another effective method for achieving high-frequency output. By selecting different gear sizes, tooth numbers, and transmission ratios, varying output speeds and frequencies can be achieved. A pair of meshing gears was introduced in a disk-shaped TENG (Fig. 8e), doubling the rotation speed and increasing current output by 36.6%, thus enhancing the collection of water and wind energy [128]. In another design, a triboelectric-electromagnetic hybrid generator based on a symmetric ratchet-gear-rack mechanism converted reciprocating motion into high-frequency rotation (Fig. 8f), resulting in current outputs of 30.94 μA and 3.76 mA, respectively [117].

Furthermore, magnetic driving has emerged as an emerging frequency-multiplication technology in recent years. Sun et al. introduced magnets and ferromagnetic blocks into TENGs, achieving different gear ratios through magnetic field modulation (Fig. 8g). Compared with the traditional FR-TENG, the charge transfer rate of this design has been increased by 4 times, and the output power of the optimized device has significantly increased by 14.6 times, reaching an average power density of 499.05 mW m⁻^2^ Hz⁻^1^ [129]. Chen et al. designed a hybrid generator with a magnetic multiplier that has the functions of frequency division and high-energy utilization efficiency. With the integrated power management circuit, thanks to the internal harmonic magnetic field coupling, the energy utilization efficiency of this generator can be as high as 93.48% [94]. In addition to the above research, Xie et al. proposed a hybrid triboelectric-electromagnetic nanogenerator inspired by magnetic gears, which has a frequency conversion effect. The TENG structure adopts a non-contact and small soft-contact mode. It shows extremely high stability. After being tested for continuous rotation for 128 h, its charge output performance did not show any significant decline [130].

Hence, while spring-driven devices are relatively straightforward to fabricate, their energy storage capacity is limited by material and design constraints, and they require regular spring replacements. Pendulum-driven systems can provide stable periodic motion but their performance is constrained by the length of the pendulum. Gear-driven mechanisms allow for precise control over frequency conversion ratios and are highly reliable, but they may encounter challenges and resistance issues under low-frequency conditions. Magnetic driving, on the other hand, is a contactless technology that offers wear-free operation and high efficiency, holding significant potential for applications in the blue energy field.

### Broadband Design

**Fig. 9:**
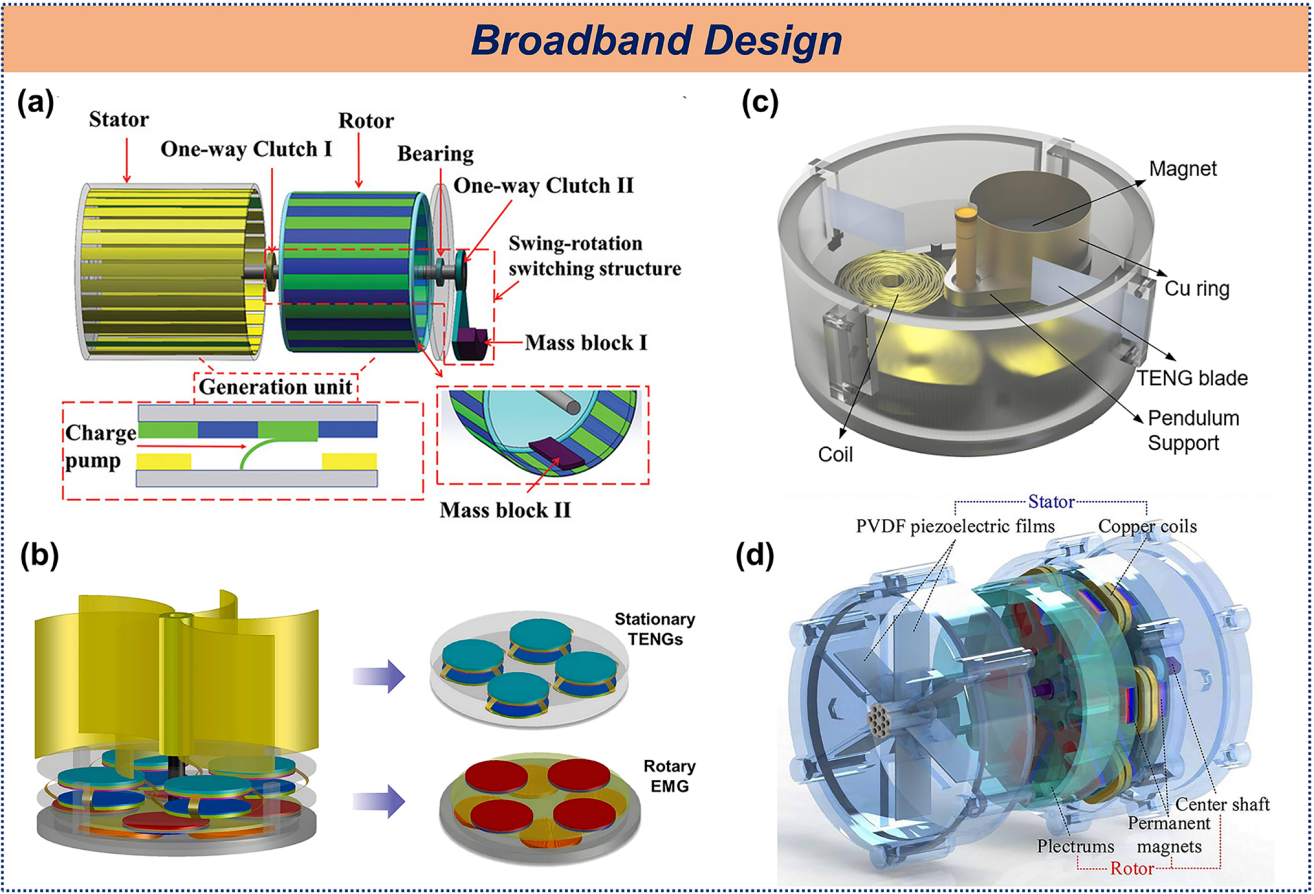
Broadband design of TENG. **a** Swing-rotation switching structure TENG. Reproduced with permission [131]. Copyright 2022, Wiley–VCH. **b** Contactless mode triggering-based ultra-robust hybridized nanogenerator, **c** rotational pendulum hybrid generator, **d** broadband rotary hybrid generator. Reproduced with permission [132–134]. Copyright 2021, 2021, 2021, Elsevier

The characteristics of ocean waves dictate that their frequency and amplitude may vary with time and location. To maximize energy-harvesting efficiency, TENGs must be capable of capturing and converting the mechanical motion of water waves across a broad frequency range to ensure continuous and stable electrical output in diverse aquatic environments. To address the issue of narrow bandwidth in cylindrical structures, a system was designed in which a pendulum swings in response to wave disturbances, storing energy through a rotational component before releasing it (Fig. 9a). This design accommodates both intermittent and continuous waves, achieving a bandwidth of 0.3–5 Hz, four times broader than that of traditional cylindrical structures [131]. In a similar approach, a dual-layer design based on a cylindrical structure was proposed, enhancing spatial utilization and enabling wideband energy collection in the 2.25–4 Hz range [88]. Hybrid generators also offer an effective method for achieving wideband energy collection. A soft magnetic coupling strategy was employed to integrate TENG with EMG (Fig. 9b), efficiently harvesting energy from water and wind flows within a range of 50–1000 rpm, ultimately creating a wireless smart farm monitoring system [132]. Another design featured a pendulum-type hybrid generator (Fig. 9c), effective for harvesting energy below 5 Hz [133], while a piezoelectric-electromagnetic hybrid generator (Fig. 9d) was developed to extend the energy collection range down to below 0.01 Hz [134].

The ability of cylindrical structures TENG to achieve wideband frequency response is fundamentally attributed to the mechanical regulation. In contrast, hybrid generators achieve wideband energy collection due to the complementary nature of TENG and EMG: TENG are suited for low-frequency energy harvesting, while EMG are more effective for high-frequency energy. This synergy between the two technologies extends the range of energy collection. The hybrid generator and mechanical gain discussed in Sects. 2.2 and 2.3 are also mentioned in this regard.

### Multi-Directional Design

External mechanical stimuli can arise from multiple directions [135]. In marine environments, mechanical energy emerges from the multi-directional motions of waves, tides, and currents. To efficiently harvest such omnidirectional energy, TENGs require specialized structural designs capable of multi-directional or omnidirectional energy collection, thereby maximizing energy-harvesting efficiency while ensuring stable power output. The core mechanism relies on spatial vector decomposition and multi-degree-of-freedom energy coupling principles. By employing isotropically responsive network or spherical architectures, the TENG can simultaneously harvest multidimensional mechanical excitations including vertical heaving (Z-axis), horizontal surge (X/Y-axes), and rotational motion (θ-direction) from ocean waves. Based on the design characteristics of TENG structures, they can be categorized into multi-directional and omnidirectional energy collection systems.

**Fig. 10:**
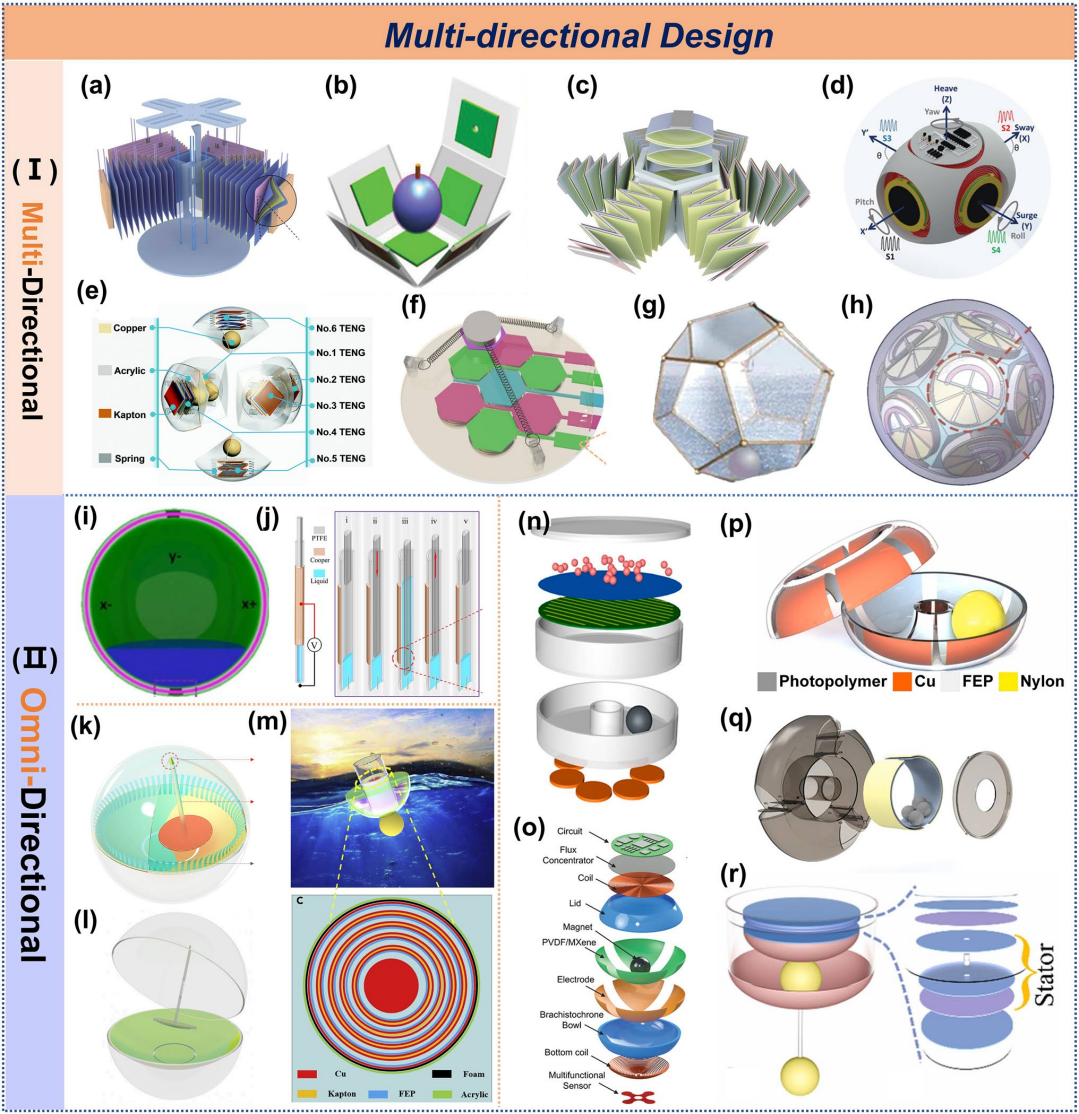
The multi-direction design of TENG. **a** Tensegrity TENG. Reproduced with permission [136]. Copyright 2023, Elsevier. **b** Multiple-frequency TENG. Reproduced with permission [137]. Copyright 2020, Wiley–VCH. **c** Flower-like TENG. Reproduced with permission [138]. Copyright 2022, Elsevier. **d** Hybridized arbitrary wave motion sensing system. Reproduced with permission [139]. Copyright 2021, Wiley–VCH. **e** Spherical TENG. Reproduced with permission [70]. Copyright 2020, The Royal Society of Chemistry. **f** Hybridized ocean wave nanogenerator, **g** multilayer wavy-structured robust. Reproduced with permission [140, [141]. Copyright 2018, 2016, Elsevier. **h** Spherical eccentric structured TENG. Reproduced with permission [87]. Copyright 2022, Wiley–VCH. **i** 3D spherical-shaped water TENG, **j** a new curvature effect TENG, **k** pendulum TENG. Reproduced with permission [142, [143, [144]. Copyright 2017, 2023, 2019, Elsevier. **l** Elastic-connection and soft-contact TENG. Reproduced with permission [145]. Copyright 2021, Wiley–VCH. **m** Active resonance TENG. Reproduced with permission [146]. Copyright 2021, Elsevier. **n** Pendulum hybrid generator. Reproduced with permission [147]. Copyright 2020, AIP. **o** Self-sustainable autonomous smart pool monitoring system. Reproduced with permission [148]. Copyright 2023, Wiley–VCH. **p** Torus structured TENG. Reproduced with permission [37]. Copyright 2019, Elsevier. **q** Barycenter self-adapting TENG. Reproduced with permission [149]. Copyright 2022, Wiley–VCH. **r** Simple fully symmetric TENG. Reproduced with permission [150]. Copyright 2023, Elsevier

Multi-directional TENGs leverage spatial arrangement of single power generation units to enhance omnidirectional energy harvesting. A representative example is the tension-modulated TENG featuring 40 units on orthogonal faces (Fig. 10a), which achieves peak output at 0° and 180° while maintaining stable performance at intermediate angles [136], consistent with earlier directional optimization principles [39]. Theoretically, the more directions a TENG covers, the more effective its energy harvesting will be. The cubic water-balloon impactor (Fig. 10b) demonstrates 28-fold enhanced charge transfer by maximizing contact area at 0° and 90° [137]. Further innovation includes the lotus-inspired six-degree-of-freedom flower-shaped TENG (Fig. 10c) capable of adapting to complex motions while delivering omnidirectional output with characteristic peaks at 60° intervals, achieving maximum values of 4.27 μC, 81 μA, and 40 V [138]. Additionally, a magnetically driven TENG was used to achieve six-dimensional wireless sensing, as shown in Fig. 10d [139]. Liang et al. upgraded the existing structure [68] by incorporating a spherical configuration with six spring-assisted units (Fig. 10e). The device demonstrates nearly identical output performance at 0° and 90°, achieving a maximum peak power of 8.8 mW, while maintaining a minimum output power of 4.0 mW at 45° [70]. The honeycomb tri-electrode design (Fig. 10f) exhibits symmetric angular response (0°-30°≈60°-90°) while enabling effective TENG-EMG hybridization [140]. Zhang et al. [141] and Qu et al. [87] expanded on the dodecahedron structure (Fig. 10g, h), providing greater potential for multi-directional wave energy collection in marine environments.

Omnidirectional energy harvesting represents the ultimate goal pursued by researchers, fundamentally distinct from multi-directional approaches through its integrated structural design philosophy. The distinctive feature of omnidirectional structures is their adaptability to water waves, primarily categorized into solid–liquid contact structures, pendulum structures, and rolling structures. The flowability of water enhances adaptability to the wave environment. Spherical symmetric designs automatically adapting to random wave directions while exhibiting characteristic output transitions between X-axis (< 45°) and Y-axis (> 45°) dominance due to contact area variations(Fig. 10i) [142]. Another design incorporated curvature effects (Fig. 10j), utilizing the periodic contact and separation of water and PTFE to generate a power output of 0.12 µW. [143]. Although water shows excellent adaptability, there is still room for improvement in its electrical output. Pendulum-based architectures address this limitation through innovative configurations including free-oscillating designs (Fig. 10k) that maintain consistent electrical output regardless of excitation angle [144]. The team subsequently optimized the structure by incorporating springs and fur shown in Fig. 10l [145]. The hemispherical pendulum structure exhibits resonant wave-matching behavior. As the tilt angle increases from 5° to 15°, its power output escalates from 7 to 12.5 mW (Fig. 10m) [146].

The third paradigm employs rolling-ball mechanisms that effectively convert random wave motion into stable electrical output, exemplified by magnetic hybrid generators (Fig. 10n) demonstrating a ninefold current reduction as angles increase from 0° to 90° [147]. Maharjan et al. designed a bowl-shaped structure in Fig. 10o [148], and Liu et al. designed a toroidal structure in Fig. 10p [37], both of which enable random wave energy harvesting through the free rolling of small balls on freestanding electrodes. Another design featured a centroid-adaptive TENG (Fig. 10q), which effectively converts water waves from all directions into rotational motion, generating electrical energy. At operating frequencies below 1 Hz, the device achieves a peak power output of 0.1 mW under a 500 MΩ load resistance [149]. Finally, a symmetric TENG (Fig. 10r) was created, employing a 360° freely rolling inertial ball and dual-layer electrodes to enable comprehensive omnidirectional wave energy harvesting. [150].

Hence, multi-direction design enhance the flexibility and adaptability of TENGs in harvesting water waves, thereby increasing their potential applications in marine energy development. These innovations offer new technological pathways to address energy demands and environmental protection.

### Hybrid Energy-Harvesting Systems

The multifunctional design of TENGs enables the integration of multiple energy-harvesting functions within a single structure, presenting significant advancements and advantages. This design effectively harnesses diverse environmental energy sources, enhances energy utilization efficiency, and reduces reliance on conventional energy sources.

**Fig. 11:**
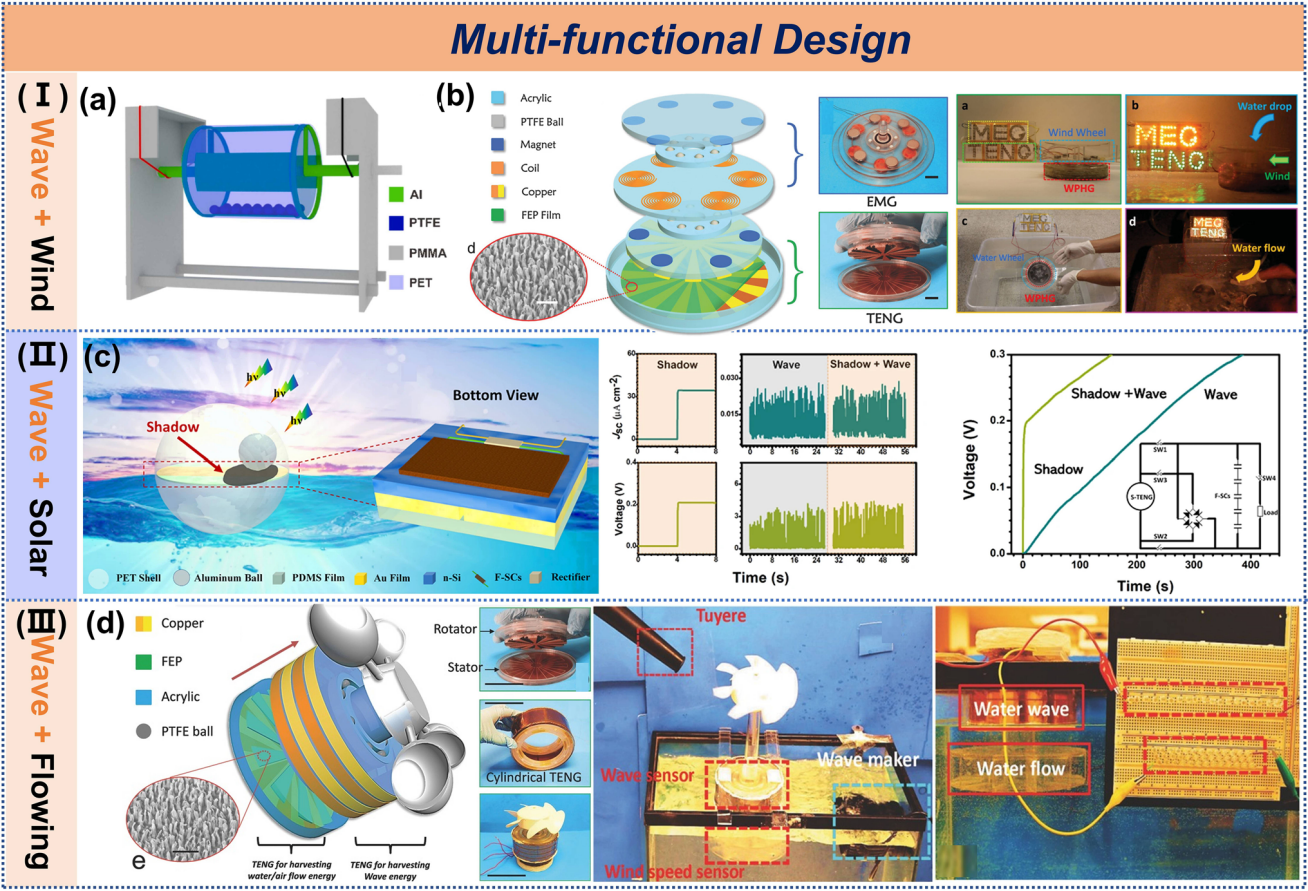
Multifunctional design of TENG. **a** Pulsed TENG. Reproduced with permission [151]. Copyright 2022, Wiley–VCH. **b** Waterproof triboelectric-electromagnetic hybrid generator. Reproduced with permission [152]. Copyright 2016, Wiley–VCH. **c** Self-charging power system. Reproduced with permission [153]. Copyright 2021, Nature Portfolio. **d** Multifunctional TENG. Reproduced with permission [154]. Copyright 2017, Wiley–VCH

To better utilize renewable resources, a cylindrical TENG was developed that is capable of simultaneously harvesting both water and wind energy (Fig. 11a), achieving a peak power density of 31 mW m⁻^2^, and successfully powering seawater electrolysis for hydrogen production [151]. In another design, a triboelectric-electromagnetic hybrid generator (Fig. 11b) used energy management through a transformer to achieve a short-circuit current of 2.3 mA, efficiently collecting both water flow and wind energy [152]. Besides wind energy, solar energy is a stable source; however, shading can affect the efficiency of solar cells. To address this, Zhang et al. integrated shading effects with triboelectric effects in a single structure (Fig. 11c), creating a self-charging power system that significantly reduced charging time to 253.3 s [153]. Furthermore, a TENG was designed to collect water wave, air flow, and water flow energy (Fig. 11d), demonstrating the ability to illuminate 50 or more LEDs in laboratory simulations [154].

The marine environment offers abundant renewable energy resources: high-density wave energy, superior wind conditions with greater consistency than terrestrial sites, and intense solar irradiation. Integrating multifunctional TENGs to simultaneously harvest these complementary energy sources presents an optimal technological solution for marine renewable energy utilization.

## Comprehensive Performance Analysis of TENGs

The above discussion outlined the structures of TENGs designed to capture water wave energy from various functional perspectives. However, the overall performance of these devices under wave action and their performance still require further analysis. First, a detailed study of the electrical output performance in different water environments is necessary. Additionally, the sustainability of the devices, including device durability and mechanical robustness, must be considered.

### Electrical Performance Analysis of TENGs

#### Analytical Models for TENG Optimization

In ocean wave energy harvesting, analytical models are crucial for multi-parameter optimization. Mathematical models and simulations are employed to refine device structural parameters, while fluid dynamics models are used to analyze wave-device interactions and hydrodynamic forces under varying ocean conditions. The synergistic integration of these approaches is critical for enhancing TENG performance and facilitating large-scale deployment.

**Fig. 12:**
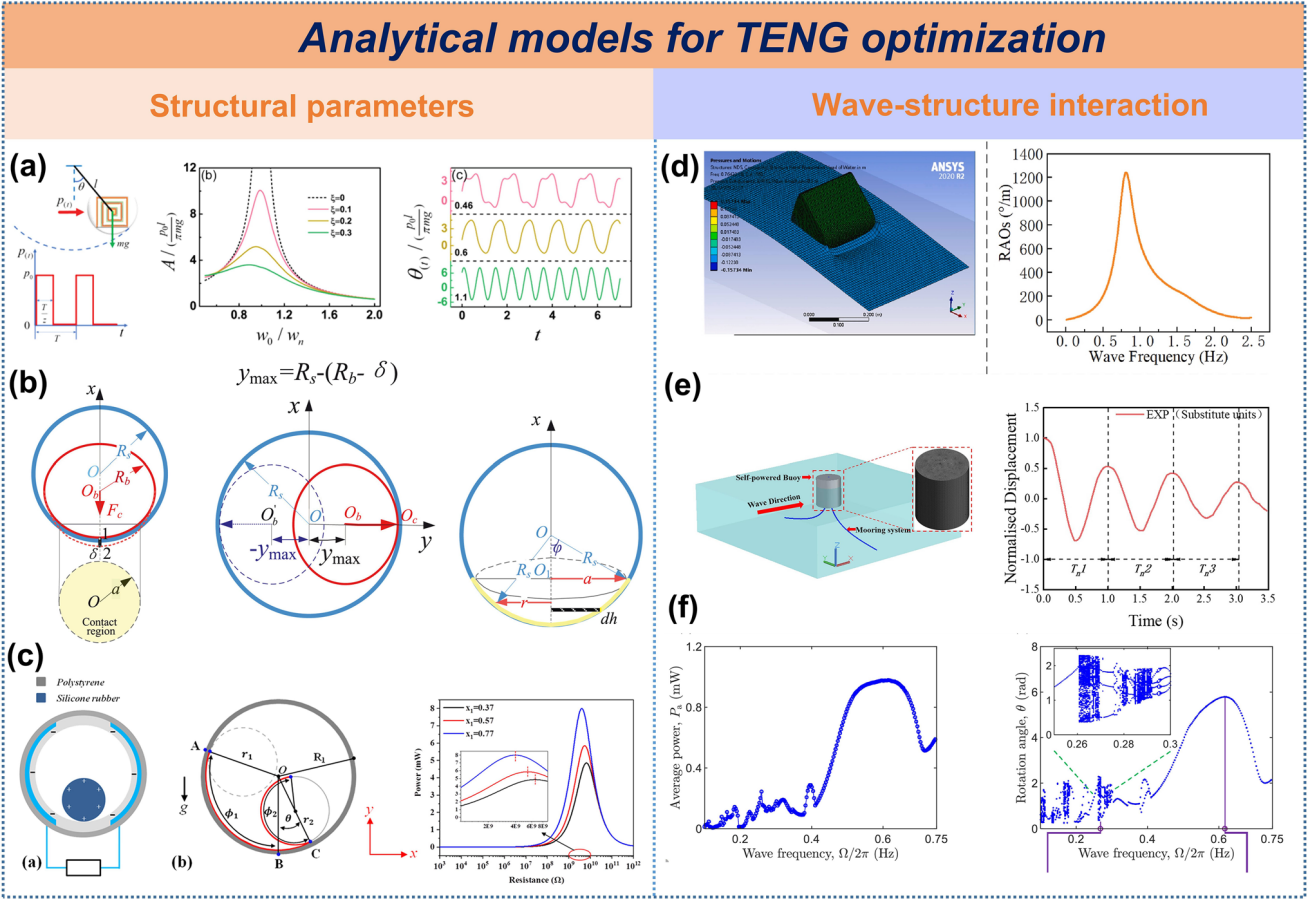
Analytical models for TENG optimization. **a** Dynamical analysis of pendulum-type TENG under unidirectional square-wave excitation. Reproduced with permission [39]. Copyright 2019, Wiley–VCH. **b** Soft sphere-shell contact characteristics, **c** solid cylinder rolls inside the cylindrical shell, **d** the simulation of the nodding duck wave energy harvester, **e** the numerical model of the self-powered buoy and its calibration and validation, **f** dynamic responses of the electret-based wave energy converter. Reproduced with permission [155, 160–163]. Copyright 2021, 2020, 2023, 2022, 2023, Elsevier

Structural Parameter Optimization: Dynamic analysis of forced vibration in pendulum-type TENGs reveals that the matching degree between external excitation frequency and the system's natural frequency significantly influences output performance. When the frequency ratio (ω_0_/ω_n_≈1), the device reaches resonance, maximizing energy output efficiency (Fig. 12a) [39]. For soft-contact spherical TENGs, the Hertz and Steuermann contact models provide effective frameworks for analyzing contact behavior between soft spheres and enclosures, though neither can fully address the transition from small-strain point contact to large conformal contact. To bridge this gap, based on numerical and analytical methods, Guan et al. established a theoretical contact electrification model on the basis of a general contact model, which can be applied to both conformal and non-conformal contact conditions. The results show that a filling rate of 83% for this structure can achieve the optimal performance(Fig. 12b) [155]. Through the evaluation of the structural quality factor, it has been found that different shell shapes have significant differences in the wave energy capture efficiency: the spherical shell exhibits the best comprehensive performance, while the cubic shell has better energy absorption characteristics in a specific direction (the Z-axis) [156]. The geometric optimization of the TENG with a regular tetrahedral structure indicates that the precise regulation of the side length ratio can maximize the power output [157]. Additionally, finite element simulations and interpolation calculations for spherical impact TENGs confirm that there exists an optimal combination of sphere size and mass parameters that can make the power output reach an extreme value [158]. Subsequently, this team also deduced based on finite element simulation and analytical equations that the stored energy can reach the maximum value when the optimal load capacitance is connected [159]. For cylinder-cylindrical shell configurations, a dynamic TENG model was established and experimentally validated, revealing that the radius ratio between inner and outer cylinders critically influences electrical output. Increasing the cylinder radius elevates peak power while reducing matched resistance(Fig. 12c) [160].

Wave-structure interaction: the ANSYS AQWA software was used to simulate the frequency-domain parameters of the buoy. A nodding duck wave energy harvester was designed in combination with the ultra-low-frequency synergy principle, and a theoretical model of the power take-off (PTO) system was established. The research results show that increasing the operating frequency and the swing angle can significantly enhance the energy output performance of the device (Fig. 12d) [161]. Numerical simulations based on 3D potential flow theory and boundary element methods indicate that buoy-type TENGs achieve optimal energy capture when mooring direction is perpendicular to wave propagation (Fig. 12e) [162]. Studies integrating linear wave theory with the Morison equation reveal that the TENG presents a nonlinear power response in regular waves and a linear relationship in irregular waves, with low-frequency superharmonic resonance effectively improving conversion efficiency (Fig. 12f) [163]. Comparative analyses of multibody dynamics and finite element simulations highlight that Salter duck-type TENGs exhibit superior performance to electromagnetic generators (EMGs) within the 2–2.75 Hz frequency range, further underscoring the deterministic role of operational frequency for both device types [77].

#### TENGs in Simulated Wave Environments

TENGs offer a promising approach to wave energy harvesting, necessitating thorough evaluation of their electrical performance. Key studies focus on output characteristics, power conversion efficiency, and hydrodynamic behavior. Current research typically involves optimizing device structural parameters using a linear motor for best performance, followed by testing the optimized device in a laboratory wave tank, and finally, implementing self-power in terminal equipment.

A comparison of the electrical outputs of the TENG devices in a hydrodynamic environment as well as in a linear motor drive is shown in Table 2. The table provides detailed performance data for each device unit under various excitation conditions and includes power conversion ratios at optimal excitation conditions for reference. Notably, the mesh design of the rolling-ball-mode TENG exemplifies a high conversion ratio. Duan et al. conducted an in-depth analysis of this design, particularly focusing on the effects of device shape and ball fill volume. Their study found that a cubic shape achieves a higher average power density of 10.08 W∙m^−3^ compared to other shapes. In a hydrodynamic environment, this structure attains a power conversion efficiency of up to 71% relative to a linear motor, highlighting its superior output and conversion efficiency [84]. Additionally, a triboelectric-electromagnetic highly coupled hybrid generator was developed based on magnetic repulsion principles, achieving a peak power conversion efficiency of 117% under water waves, despite the dielectric shielding effect of water, showcasing its exceptional underwater adaptability [38]. This underscores the significant research potential of magnetic-assisted structures in hydrodynamic power generation, though reports on this are still limited. Furthermore, designs featuring a book-shaped structure [36], a spring-assisted structure [69], and a gas-assisted structure [62] achieve power conversion rates of 77%, 80%, and 100%, respectively, in simulated water waves. These designs feature multilayer TENG structures on flexible substrates that effectively harness longitudinal wave frequencies and amplitudes to generate charge.

**Table 2:** Comparison of electrical output of TENGs under motor and water waves

Device structure	Module		Motor Condition					Water Environment				*Power Conversion Percentage	Refs
			Motor Excitation	Matching Impedance		Power/Power Density		Water Frequency	Water Amplitude	Matching Impedance	Power /Power Density		
Cube	TENG		2 Hz	500 MΩ		2.95 mW		0.8 Hz	6 cm	300 MΩ	2.10 mW	71%	[84]
Pendulum	TENG		1 Hz	200 MΩ		1.51 mW		0.4 Hz	2.5 V	200 MΩ	0.61 mW	40%	[79]
Swing	TENG		7.5 m s^−2^	300 MΩ		0.14 W m^−3^		1.2 Hz	10 cm	50 MΩ	26.3 mW m^−3^	19%	[45]
Spherical	TENG		0.6 Hz	500 Ω		32.1 mW		0.6 Hz	6 V	300 Ω	25.8 mW	80%	[69]
Arc	TENG		15 m s^−2^	30 MΩ		0.7 mW		1.2 Hz	8.5 cm	40 MΩ	0.3 mW	43%	[165]
Swing	TENG		8 m s^−2^	25 MΩ		9.6 mW		1.1 Hz	10 cm	3 MΩ	6.2 mW	65%	[46]
Magnetic- Levitation	TENG		8 m s^−2^	10 MΩ		7.10 mW		1 Hz	8 cm	10 MΩ	8.21 mW	117%	[38]
	EMG			8 kΩ		0.47 mW				8 kΩ	0.12 mW	26%	
TPU-assisted	TENG		8 m s^−2^	500 kΩ		42.68 mW		1.1 Hz	8 cm	4 MΩ	7.44 mW	17%	[61]
	EMG			40 kΩ		4.40 mW				40 kΩ	0.10 mW	2%	
Butterfly	TENG		5 m s^−2^	20 MΩ		28.125 mW		1.25 Hz	/	20 MΩ	11.87 mW	42%	[55]
	TENG		5 m s^−2^	20 MΩ		83.205 mW		1 Hz	/	15 MΩ	18.509 mW	22%	
Open-book	TENG		0.8 Hz	13.8 MΩ		9.675 W m^−3^		0.588 Hz	/	13.8 MΩ	7.45 W m^−3^	77%	[166]
Self-assembly	TENG		1.67 Hz	1 GΩ		8.69 W m^−3^		1.45 Hz	/	50 MΩ	2.05 W m^−3^	24%	[83]
Cylindrical	TENG		0.33 Hz	70 MΩ		0.69 W m^−3^		0.33 Hz	/	70 MΩ	0.49 W m^−3^	71%	[127]
Pendulum	TENG		1.75 Hz	20 MΩ		5.2 mW		0.8 Hz	/	20 MΩ	0.87 mW	17%	[167]
Cylindrical	TENG		1.75 Hz	560 kΩ		14.357 mW		0.67 Hz	/	560 kΩ	7.07 mW	49%	[164]
Multi-layered	TENG		1 Hz	30 MΩ		27.8 W m^−3^ Hz^−1^		1.1 Hz	/	5 MΩ	3.64 W m^−3^ Hz^−1^	13%	[73]
Rolling	TENG		100 rpm	/		15.0 mW		0.8 Hz	100 mm	4 MΩ	7.6 mW	51%	[168]
Swing	TENG		2.0 Hz	/		53.5 mW		0.8 Hz	7.0 cm	5 MΩ	27.6 mW	52%	[47]
Swing	TENG		/	/		7.56 mW		0.7 Hz	10 cm	10 MΩ	4.27 mW	56%	[169]
Spherical	TENG		1 Hz	/		12.1 mW		1 Hz	10 cm	10 MΩ	4.1 mW	34%	[56]
Pendulum	TENG		20°	1 MΩ		39.2 mW		/	/	20 MΩ	3.1 mW	8%	[74]
	PENG			700 Ω		30 mW				2 kΩ	2.9 mW	10%	
	EMG			500 kΩ		2.5 mW				1 kΩ	0.6 mW	24%	
Spherical	TENG		1.0 Hz	50 MΩ		79 W m^−3^		/	/	20 MΩ	26.2 W m^−3^	37%	[99]
Pendulum	TENG		1.0 Hz	5 MΩ		20.1 mW		1.0 Hz	10 cm	5 MΩ	0.845 mW	/	[170]
Multi-layered	TENG		2.5 Hz	/		5.5 mW		1.0 Hz	9.0 cm	10 MΩ	2.5 MΩ	45%	[60]
Gas-assisted	TENG		/	20 MΩ		3.0 mW		1 Hz	/	/	3.0 mW	100%	[62]
Anaconda	TENG		20°	5 MΩ		347 W m^−3^		/	/	5 MΩ	80.61 W m^−3^	23%	[44]
Pendulum	TENG		20°	5 MΩ		200 W m^−3^		/	/	2 MΩ	34.7 W m^−3^	23%	[57]
Pendulum	TENG		4 Hz	100 MΩ		0.15 mW		1.0 Hz	/	/	0.023 mW	15%	[112]
	EMG			60 Ω		4.7 mW				/	0.57 mW	12%	
Pendulum	TENG		1.5 Hz	5 MΩ		12.3 mW kg^−1^		/	/	/	6 mW kg^−1^	49%	[86]
	EMG			500 Ω		6.6 mW kg^−1^				/	0.15 mW kg^−1^	2%	
Pendulum	TENG		2.6 Hz	10 MΩ		16 mW		1.4 Hz	10.2 cm	/	7.3 mW	46%	[75]
	TENG			50 MΩ		55 μW		1.2 Hz		/	30.3 μW	60%	
	EMG			7 kΩ		0.16 mW		1.4 Hz		7 kΩ	0.07 mW	44%	
Shuttle	TENG		5 m s^−2^	/		9.0 mW		0.8 Hz	/	3 MΩ	3.6 mW	40%	[121]
	PENG			/		26.2 mW				4 KΩ	3.6 mW	14%	
	EMG			/		34.2 mW				1.3 KΩ	6.3 mW	18%	
Pendulum	TENG		15°	/		12.5 mW		/	/	5 MΩ	12.3 mW	98%	[146]

^*^ The power conversion percentage indicated by the asterisk is derived from the optimal power (or power density) obtained from motor-driven devices in air, as reported in the corresponding literature, compared to the optimal power achieved under simulated aquatic conditions in the laboratory

However, the Kapton-based multilayer TENG structures designed by Li et al. [73] and Zhang et al. [57, 74] exhibit relatively low power conversion efficiencies in water, ranging from 13 to 17% and 8%, respectively. While these multilayer designs deliver commendable power output under motor driving, their efficiency in water remains suboptimal due to the constrained motion of units in directions other than the driven axis, necessitating further optimization. Additionally, the power conversion efficiency of triboelectric-electromagnetic hybrid generators was also evaluated. In the designs by Wang et al. [61] and Zhang et al. [86], the TENGs achieve conversion efficiencies of 17% and 49%, respectively, while the EMGs show a conversion rate of only 2%. This indicates that TENGs are more suited for capturing low-frequency water waves, whereas EMGs are better for high-frequency waves. Future research should focus on optimizing the synergy between these two technologies.

**Fig. 13:**
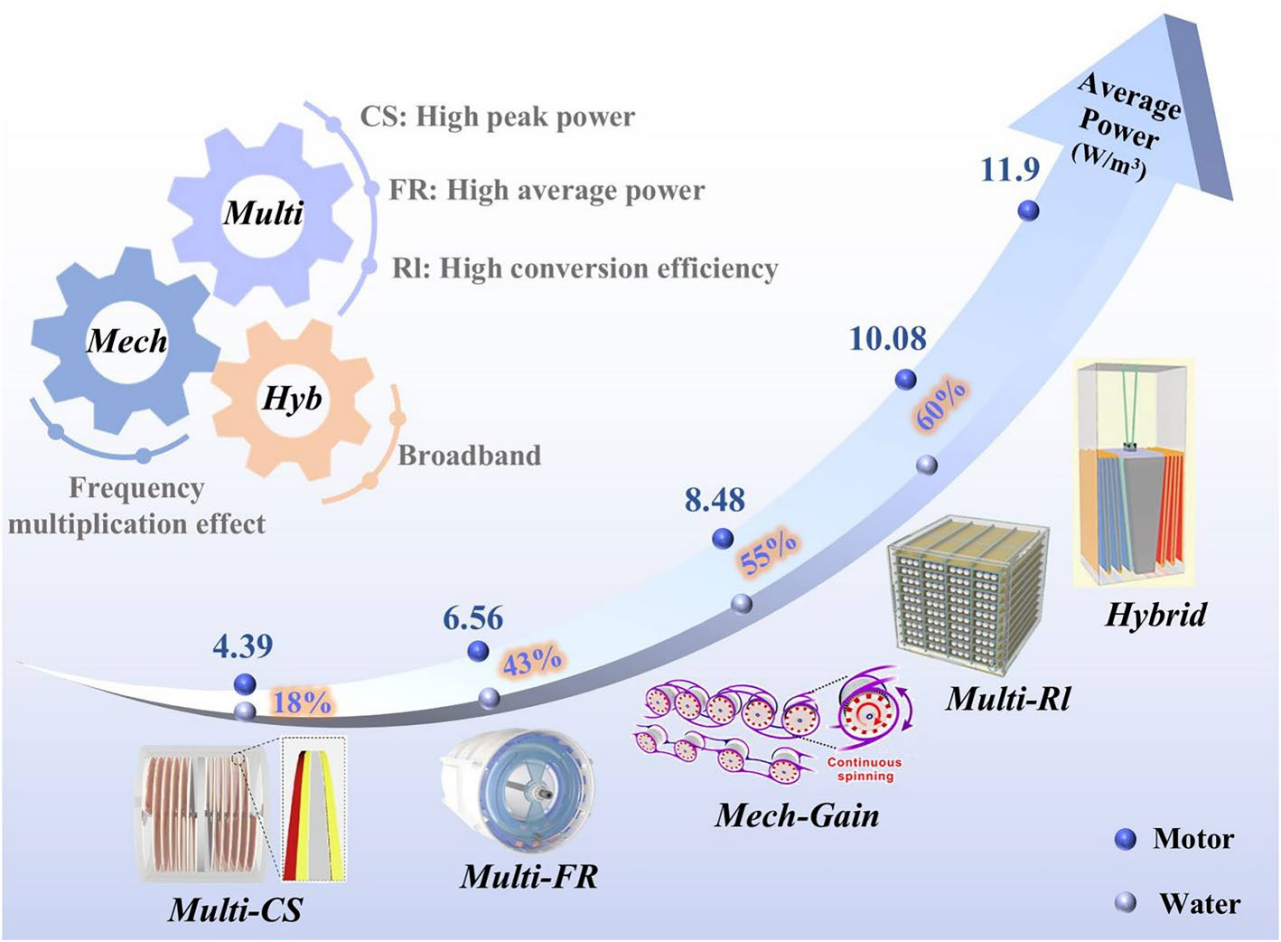
Average power of advanced TENGs and power conversion in water environments and motor. Reproduced with permission [44]. Copyright 2022, Elsevier. Reproduced with permission [47, 74]. Copyright 2023, 2022, Wiley–VCH. Reproduced with permission [164]. Copyright 2023, The Royal Society of Chemistry. Reproduced with permission [84]. Copyright 2023, Springer (Here, "Muti" represents multilayer design, “Mech” and “Mech-Gain” denote mechanical gain design, “Hyb” and “Hybrid,” respectively, signify hybrid generator. Additionally, “Muti-CS”, “Muti-FR” and “Muti-Rl,” respectively, stand for TENG in contact-separation mode, freestanding mode and rolling mode within the multilayer design.)

Overall, most current devices exhibit satisfactory power conversion efficiencies in water, generally achieving over 40%. The design features discussed earlier, such as high space utilization, hybrid structures, and mechanical gain, primarily aim to enhance the electrical output of the devices. Figure 13 displays the most representative results under different functional design. Each of the different operating modes has its own unique advantages. The traditional contact-separation mode can exhibit a high peak density, which is of great significance in certain specific demand scenarios. Meanwhile, the freestanding mode and the rolling mode perform more prominently in terms of average power and conversion efficiency. Especially for the rolling mode, its power conversion efficiency can be as high as 60%, and its average power also reaches an impressive 6.02 W m^−3^. This structure thus holds significant commercial potential. Moreover, the frequency-multiplication effect brought about by mechanical gain provides an effective way to increase the average power. The hybrid generator also has good performance. It can achieve relatively ideal electrical output by effectively capturing water waves in a wider frequency band. To better adapt to real marine environments, it is recommended to optimize device structural parameters in simulated water waves. This approach aligns more closely with the actual motion dynamics of devices, as motor motion is limited to horizontal and vertical linear movements, while real marine motion results from the combined effects of horizontal and vertical water waves. Additionally, it is essential to conduct targeted testing of TENG electrical properties across different frequencies and amplitudes of water waves to better understand the device's response to varying wave conditions.

Building on the functionalized structural design of TENGs, scalable array configurations are crucial for industrial implementation. Current research focuses on four primary array architectures: parallel, tandem, grid, and three-dimensional (3D) arrays, s, each with distinct advantages for marine energy applications. Parallel arrays enable significant planar scalability. Arc-shaped arrays have achieved a peak power density of 2.34 W m^−3^ [165], while a 38-unit array delivered 15 μC charge and 13.23 W m^−3^ peak power [171]. Tandem arrays are optimized for confined spaces with linear or chain-like setups. Qiu et al., inspired by Brownian motion, achieved unidirectional rotation of a TENG chain array using an inertial wheel in Fig. 15g [164]. Grid arrays (2D) maximize area coverage through mesh arrangements, creating dense structures suitable for high-density energy harvesting. Researchers have arranged TENG units in 2 × 2, 3 × 3, and 4 × 4 square grid arrays for temperature sensing [37, 56, 172]. Three-dimensional arrays show the most promise for commercialization, combining high spatial efficiency with enhanced energy capture. A hexagonal, seven-layer sandwich structure powered 12W LEDs [173], with modeling predicting a 61.20 mW peak output, underscoring their superior space utilization and cost-effectiveness [162].

Large-scale wave-making facilities have become essential for experimental validation. Xu et al. tested hexagonal S-TENG buoy arrays in a 50 m × 3 m × 1 m wave basin, optimizing performance at the resonance between wave and buoy frequencies, achieving a short—circuit current of 20.91 μA and transferred charge of 2.22 μC [173]. Another innovation integrated oscillating water column (OWC) technology with TENGs. After optimization in a 45 m × 4 m × 3 m towing tank, a 1m-high cylindrical device (0.6 m diameter) generated 55.45 μA current and 5.28 mW power (114.8 W m^−3^ power density) [174]. Subsequent hybrid systems incorporating electromagnetic generators further improved performance, demonstrating the superiority of combined configurations [175].

#### TENGs in Real-Water

Research on TENG for harvesting water wave energy involves not only laboratory-based wave simulation tests but also crucial real-sea electrical output assessments. Such field tests effectively validate the TENG's performance and reliability in actual marine environments. The tests in the real sea can more accurately assess the stability and durability of the TENG in long-term operation, as well as the efficiency of the electrical output under actual operating conditions. These insights are essential for optimizing the TENG's design and provide necessary technical support and market competitiveness for commercial applications.

**Fig. 14:**
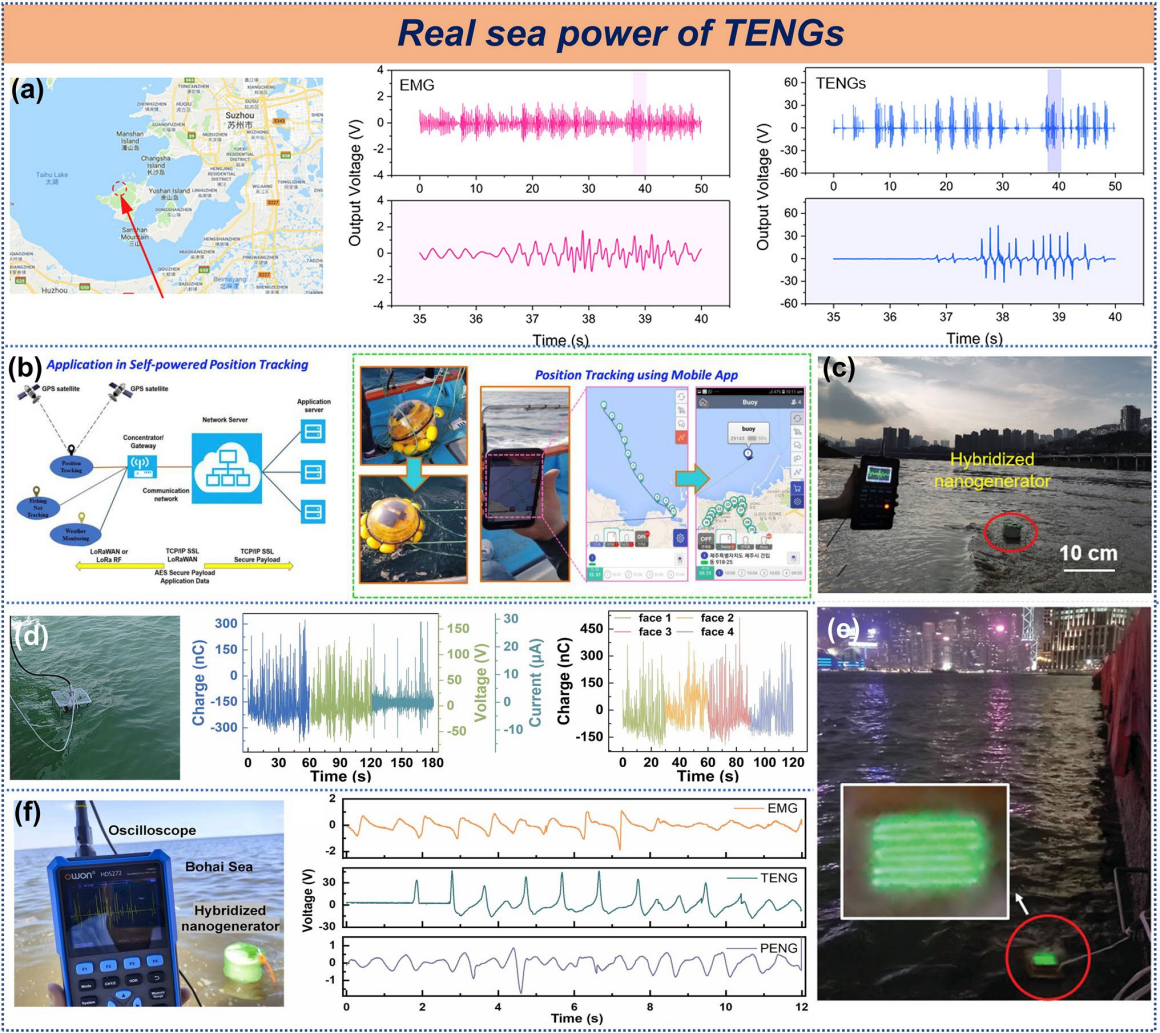
Real-sea power of TENG. **a** Hybrid generator for energy scavenging in real water, **b** real-time application of SB-HG, **c** real-time testing of hybridized nanogenerator in Jialing River, **d** T-TENG in the real marine environment. Reproduced with permission [97, 108, 133, 136]. Copyright 2019, 2020, 2020, 2023, Elsevier. **e** LED array powered by water waves in Victoria Harbor. Reproduced with permission [176]. Copyright 2021, Wiley–VCH. **f** Output performance of the SPC-HEH in real water. Reproduced with permission [40]. Copyright 2024, Elsevier

Several studies have reported field tests of TENGs in real marine environments. For instance, a rotating pendulum triboelectric-electromagnetic hybrid nanogenerator was tested in Taihu Lake (Fig. 14a), achieving voltages of 80 V for TENG and 3.4 V for EMG, regardless of its orientation [133]. A spherical triboelectric-electromagnetic hybrid nanogenerator was also demonstrated successfully, charging a lithium-ion battery and providing real-time tracking of a buoy's position (Fig. 14b), even when several kilometers offshore [97]. In Fig. 14c, testing on the Jialing River revealed that a chaotic pendulum triboelectric-electromagnetic hybrid nanogenerator responded more effectively to random environments compared to linear systems [108]. In another study, a high-density stacked TENG with a tension structure was tested under real marine conditions (Fig. 14d), showing excellent energy-harvesting performance across all wave directions [136]. In Fig. 14e, Wu et al. used a high volumetric charge density solid–liquid contact TENG to light 150 LEDs in Victoria Harbour, Hong Kong [176]. A triboelectric-magnetic-piezoelectric hybrid nanogenerator tested in the Bohai Sea achieved peak voltages of 46, 1.9, and 1.7 V (Fig. 14f), demonstrating significant potential for water wave energy harvesting [40]. Additionally, reliability validations were conducted by Zhou et al. [177], Wu et al. [92], Yang et al. [83], and Zhai et al. [121] in real marine environments.

Overall, field tests conducted in real marine environments demonstrate that TENGs possess significant potential and readiness for sustainable development in the blue energy, including energy harvesting and conversion.

### Sustainability Performance of TENGs

#### Device Durability

The durability of TENGs is crucial for reliable wave energy harvesting. Systematic durability assessments reveal performance degradation patterns and guide design optimization for marine applications. Our analysis of various TENG structures identifies two key durability factors: material wear and fatigue and mechanical design.

**Fig. 15:**
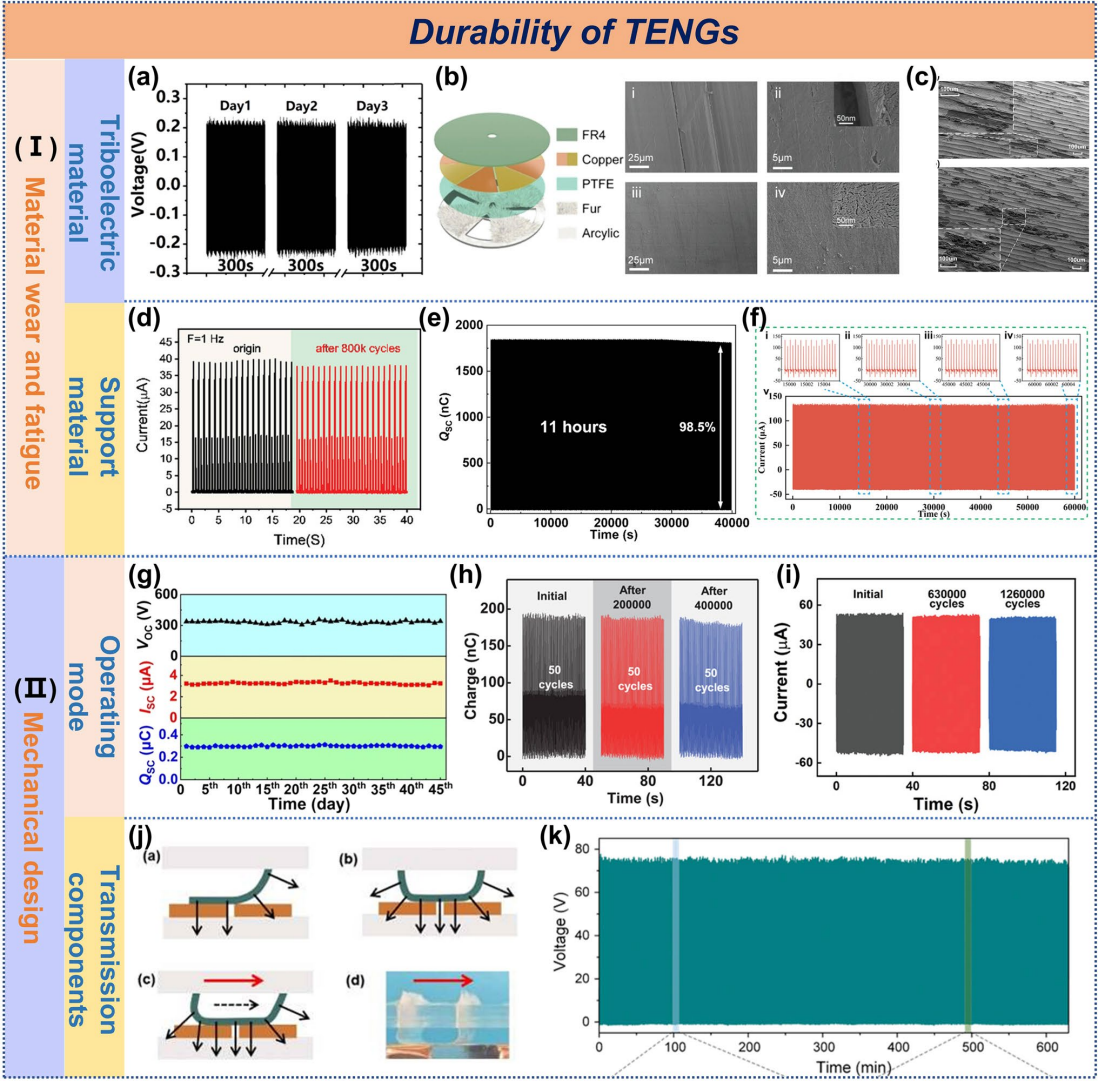
Durability of TENGs. **a** Durability of WS-TENG tested for three days. Reproduced with permission [182]. Copyright 2019, Elsevier. **b** SEM images contrasting PTFE film surfaces worn by Cu and rabbit fur. Reproduced with permission [128]. Copyright 2021, Wiley–VCH. **c** SEM images of FEP film after 3-month operation with PA and without PA. Reproduced with permission [116]. Copyright 2023, Elsevier. **d** Durability of zigzag multi-layered TENG. Reproduced with permission [59]. Copyright 2019, Wiley–VCH. **e** Durability of the GA-TENG. Reproduced with permission [62]. Copyright 2024, Elsevier. **f** Stability of ML-TENG. Reproduced with permission [38]. Copyright 2024, Wiley–VCH. **g** Durability test for the BBW-TENG within 45 days. Reproduced with permission [82]. Copyright 2022, Elsevier. Durability of the **h** SS-TENG and **i** RS-TENG at various cycles. **j** Mechanical study of different TENG, durability test and **k** output power of the ES-TENG. Reproduced with permission [45, 86, 145, 169]. Copyright 2020, 2023, 2023, 2021, Wiley–VCH

*4.2.1.1 Material wear and fatigue: Regarding triboelectric materials:* Minimizing inter-material wear significantly enhances device durability [178–180]. In terms of contact modes, solid–liquid contact TENGs exhibit far less wear than solid–solid contact counterparts [181]. By avoiding direct mechanical friction between solid tribo-layers, solid–liquid contact significantly reduces material fatigue and surface microstructure damage. For instance, a curvature-based solid–liquid TENG employing PTFE rods in contact with water maintained a stable open-circuit voltage of 25 V for 3,600 s without noticeable attenuation [143]. Additionally, liquid–solid interfacial TENG wave sensor developed by Xu et al. maintained stable output voltage and preserved PTFE surface microstructure after continuous operation for three days (Fig. 15a) [182]. Hydrophobic modification of the solid tribo-layer in solid–liquid TENGs can further enhance both electrical output and long-term stability [183].

In contrast, solid–solid contact TENGs show greater durability dependence on tribo-material selection. Chen et al.'s study revealed that direct PTFE-copper (Cu) contact friction caused severe PTFE surface scratching after 72,000 cycles, leading to a 20.1% output decline [128]. This degradation primarily resulted from PTFE wear debris adhering to Cu surfaces, reducing surface potential. When rabbit fur replaced Cu as the tribo-material, PTFE surfaces remained nearly undamaged due to fur's high density and softness, with surface morphology even becoming smoother than pristine states (Fig. 15b). Another study comparing FEP (negative layer) paired with either nylon (PA) or Cu (positive layer) demonstrated that PA-FEP configurations maintained 98% voltage stability after 100k rotation cycles, outperforming Cu-FEP combinations (91%). After three months of repeated use, FEP films without PA dielectric interlayers exhibited more scratches and protrusions, confirming the important role of the lubricious PA film in improving the stability of TENGs (Fig. 15c) [116].

*Regarding support materials*: Depending on functional requirements, common supporting materials include origami structures, polymer films (e.g., PET, Kapton), 3D-printed flexible scaffolds (e.g., TPU), spring steel sheets, and magnetic frameworks. Studies (Fig. 15d) show that paper-based pendulum hybrid nanogenerators experienced measurable rectified short-circuit current decay after 800 k cycles [59]. For the U-shaped TENG supported by PET, after 45 k cycles, the short-circuit charge Q_sc_ decreased from 745 to 699 nC, with an attenuation rate of approximately 6.17% [184]. In contrast, gas-assisted TENGs with 3D-printed TPU skeleton maintained 98.5% initial performance after 11 h of simulated wave testing(Fig. 15e) [62]. Spring steel stands out for its exceptional mechanical strength and elastic recovery. The multi-arch array TENGs using spring steel maintained stable outputs over 14 h of continuous operation, showing negligible degradation even after 3-day endurance tests [65]. Notably, NdFeB-based magnetic support structures eliminate mechanical wear entirely through contactless magnetic levitation. A frequency-multiplication magnetic-levitation hybrid generator demonstrated highly stable fundamental and harmonic current outputs over 60,000 s(Fig. 15f) [38]. Given permanent magnets' theoretically infinite lifespan, such designs offer unparalleled long-term stability. The work of Zhang et al. [67] and Lou et al. [66] provides compelling evidence for the merits of magnetic support.

In brief, the durability of solid–liquid contact TENGs can withstand more rigorous tests. For solid–solid TENGs, incorporating soft, high-density fur materials effectively minimizes tribo-layer wear, while lubricating films (e.g., PA) mitigate surface microstructure damage, enhancing output stability. For support materials, their durability has an obvious correlation with their mechanical properties. Although polymer materials are low in cost and easy to process, they are prone to fatigue damage under long-term dynamic loads; 3D-printed flexible skeletons perform well within a moderate service cycle; while spring steel sheets and magnetic force support structures exhibit better long-term stability. Practical engineering applications require balanced consideration of operational environment, cost, and lifespan to optimize the trade-offs among structural complexity, economy, and durability.

*4.2.1.2 Mechanical design: Operating mode*:In the mechanical design of TENGs for water wave energy harvesting, the selection of operational modes exhibits a significant correlation with device durability. Based on the contact patterns at the triboelectric interface, these modes can be systematically categorized into four types: non-contact, point contact, line contact, and surface contact, each with distinct durability performance.

Studies demonstrate that non-contact designs exhibit superior durability. A multilayer swing-structured TENG, employing a non-contact mode, maintained stable output current after 240,000 cycles, with SEM characterization revealing no significant wear on the Kapton film surface [47]. Sun et al. demonstrated that non-contact operation between rotor and stator in freestanding TENGs yields significantly lower charge retention than direct-contact modes [185]. Similarly, a pendulum-inspired freestanding TENG, incorporating a 1 mm gap between the triboelectric layer and electrode, retained stable electrical output after 1,000,000 cycles, with SEM confirming the intact nanostructure of the PTFE film [144]. These findings highlight the superiority of non-contact modes in preventing mechanical wear. Additionally, a bioinspired butterfly-wing TENG utilizing point contact via PTFE ball rolling maintained stable output after 45 days of continuous testing in water environments (Fig. 15g) [82]. The durability of rolling point-contact modes was further validated by tower-shaped [78] and soft bioinspired fin-structured TENGs [113]. A swing-structured TENG designed by Jiang et al., employing surface contact with flexible PTFE brushes, retained consistent charge transfer after 400,000 cycles (Fig. 15h) [45]. When the surface-contact PTFE brushes were replaced with roller-based line contact, the device only exhibited only a 1.6% performance degradation after 1,260,000 cycles (Fig. 15i) [169].

These results establish a clear correlation between TENG operational modes and durability, with the following ranking: non-contact > point contact > line contact > surface contact. This trend arises from tribological differences among contact modes: non-contact modes theoretically achieve near-zero wear by eliminating mechanical interaction; point contact minimizes frictional losses by restricting the contact area; line contact accumulates wear due to increased contact area; while surface contact induces pronounced friction-induced damage. Consequently, the design of TENG operational modes must strike an optimal balance between energy conversion efficiency and durability based on specific application requirements.

*Transmission components*: In the mechanical design of TENGs, the rational application of transmission structures plays a pivotal role in achieving mechanical gain effects. Common transmission components such as gears, springs, and pendulums exhibit distinct characteristics in terms of durability. The gear-based double-rocker structure TENG developed by Yang et al. maintained stable output performance after 100,000 cycles of testing [126]. Zhang et al. developed a gear-driven wave energy-harvesting US-TENG with a low frictional torque of 2.7 N cm. After 100,000 cycles, it maintained 60% of its original output, far exceeding the performance of planar-contact TENGs that degraded to below 10% after only 30,000 cycles (Fig. 15j) [86]. In the ratchet-gear-rack hybrid power generation system developed by Cheng et al., the TENG component exhibited an 88% retention of short-circuit current after 100,000 cycles [117]. Compared to gear transmissions, pendulum structures demonstrate superior durability. Lin's team optimized a pendulum mechanism through elastic connections and soft-contact design [145], enabling stable output even after 2 million cycles—a marked improvement over the original design's 1-million-cycle lifespan [144] (Fig. 15k). This enhancement primarily stems from effective friction reduction during motion. Furthermore, Liang et al. integrated spring energy storage with pendulum reciprocation to design a spherical TENG, further validating the stability of hybrid structures in energy conversion [56].

From a durability perspective, gear transmissions, while reliable for power transfer and frequency conversion, are susceptible to material wear and lubrication conditions, requiring regular maintenance—especially in harsh environments. Spring components, though highly durable, are prone to fatigue failure under high-frequency loads and necessitate special alloy materials to extend service life. In contrast, pendulum structures exhibit the best performance due to minimal frictional losses, though pivot bearing wear remains an issue that must be mitigated through low-friction material selection. It is worth emphasizing that the durability of transmission structures depends not only on component type but also critically on material properties, lubrication conditions, and contact mechanisms.

#### Mechanical Robustness

The durability of TENGs primarily examines their performance stability under long-term operational conditions, while mechanical robustness focuses on their resistance to degradation in complex physical environments. Given Earth's extreme environmental variations—from polar regions (-70 ℃) to deserts (60 ℃), and from 0% RH (arid zones) to 100% RH (tropical rainforests)—adaptability to such fluctuations is critical for reliable operation in harsh marine settings [186]. This directly impacts device service life and safety. Key external environmental factors include climatic conditions such as temperature (T), relative humidity (RH), and ultraviolet (UV) radiation, as well as chemical environments like pH, salinity (S), and ion concentration ([X]). These factors interfere with the surface charge transfer processes of materials, thereby affecting electrical output performance.

**Fig. 16:**
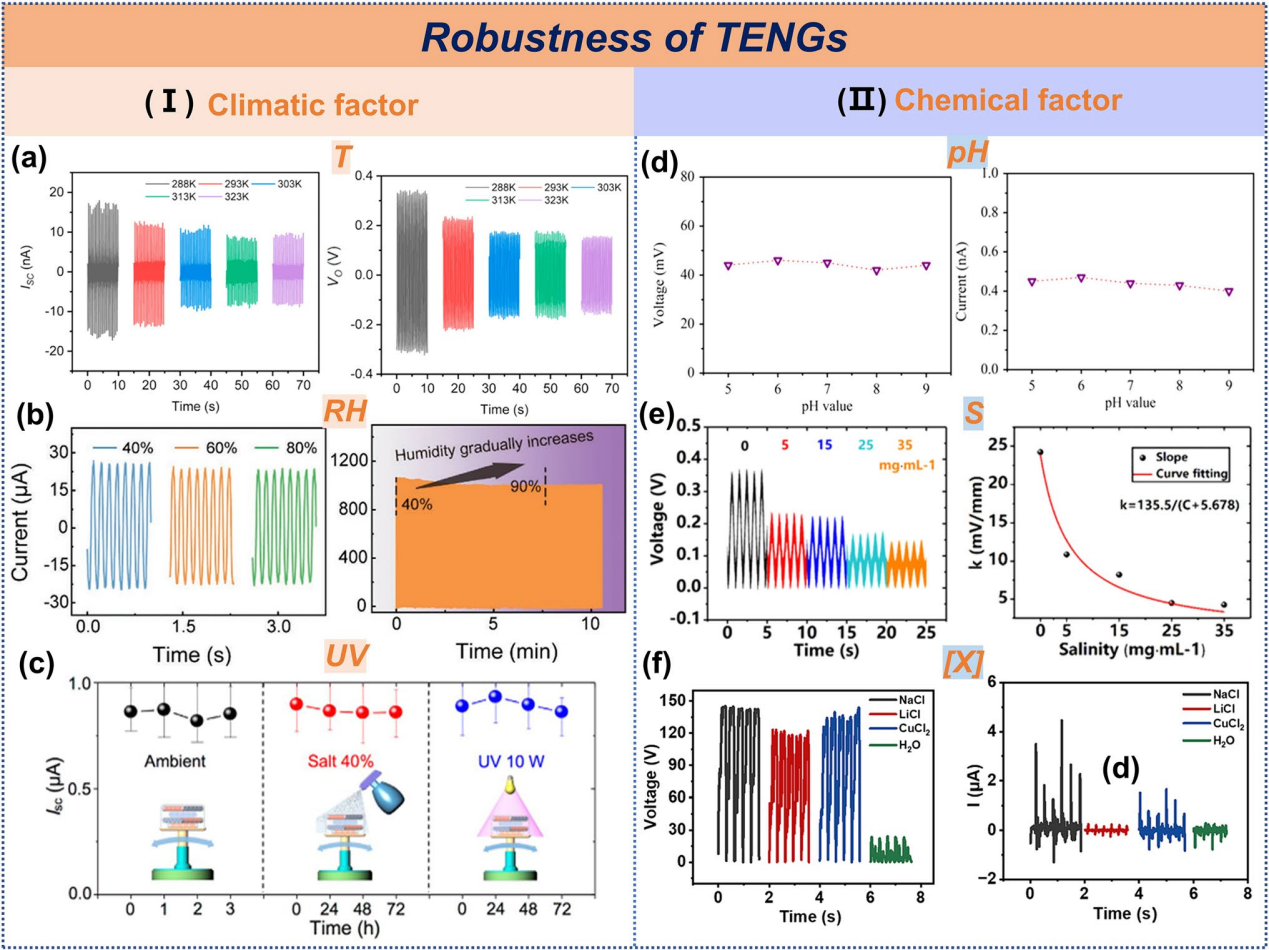
Robustness of TENGs. **a** Effect of temperature on the triboelectrification at the solid–liquid interface. Reproduced with permission [187]. Copyright 2020, Elsevier. **b** Effect of humidity on the charges of TENG. Reproduced with permission [128]. Copyright 2021, Wiley–VCH. **c** Comparison of short-circuit currents (I_sc_) under different environment, **d** output of TENG at different pH values of water, **e** the impact of salinity on the WS-TENG, **f** outputof the SSEP-TENG with different salt solutions. Reproduced with permission [182, 189–191]. Copyright 2018, 2016, 2019, 2024, Elsevier

Environmental factors such as temperature, humidity, and UV radiation significantly influence the performance of TENGs. Zhang et al. systematically studied temperature-dependent triboelectric behavior at solid–liquid interfaces using a tubular TENG, revealing decreased electrical output with rising temperature (Fig. 16a) [187].In extreme temperature adaptation, Jung et al. developed an Arctic-TENG capable of delivering a 5 μA higher current at -40 °C than at room temperature, demonstrating exceptional temperature resilience [188]. For humidity adaptability, Chen et al. designed a rabbit fur-brush-based TENG that maintained stable electrical performance for over 10 min within a humidity range of 40%–90% (Fig. 16b) [128]. Additionally, encapsulation technology plays a critical role in enhancing robustness. Guo et al. achieved long-term stable operation of a triboelectric-electromagnetic hybrid generator in high-humidity environments through magnetic non-contact encapsulation [152]. Under strong UV radiation in marine settings, Kim et al. reported that a buoy-based TENG exhibited stable performance after 72 h of exposure to 10W UV radiation, benefiting from the protective effect of acrylic fillers (Fig. 16c) [189].

Compared to solid–solid contact TENGs, solid–liquid contact TENGs are more significantly influenced by the pH, salinity, and ion composition of the liquid environment. Regarding pH, Liang et al. demonstrated that a transparent TENG exhibited minimal variation in current and voltage within the pH range of 4–9, indicating good acid–base adaptability (Fig. 16d) [190]. However, Zhang et al. demonstrated that a more acidic environment is detrimental to the electrical output [187]. Gao et al. further explained this phenomenon, stating that the increase in hydrogen ion concentration promotes the generation of cations, thus suppressing the electrostatic induction on the surface of the negative triboelectric layer [116]. Concerning salinity, Xu et al. observed that as the salinity of NaCl solution increased from 0 to 35 mg mL^−1^, the voltage of TENG decreased by 57%, showing a linear trend due to high ion concentration impeding electron transfer at the solid–liquid interface (Fig. 16e) [182]. In contrast, Zhao et al. explored the effects of ionic solutions with different valences on the performance of TENGs and discovered that the NaCl solution was less affected by external factors compared to other solutions (Fig. 16f) [191].

Nevertheless, current TENGs still face significant challenges in robustness. In complex environments such as the ocean, the synergistic effects of high salinity, strong acid/base conditions, and UV radiation accelerate material degradation and reduce electrical output stability. Although progress has been made in specific environmental adaptability studies, existing solutions often lack systematic and universal applicability, making it difficult to meet the demands of diverse application scenarios. This severely limits their large-scale practical deployment. Future research should focus on developing materials and structural design strategies with broad-spectrum environmental adaptability.

## Conclusions and Prospects

In the realm of marine energy, TENGs for harvesting ocean wave energy hold immense potential. While previous studies have extensively examined material optimization, energy management, and applications of TENG devices, this review focuses on the functional structural design, underwater output performance, and device sustainability, with other related areas not being elaborated upon.

### Conclusions

The invention of lightweight and cost-effective TENGs presents a remarkable opportunity to harness the widely distributed and high-energy–density blue energy resources. To accelerate the commercial application of TENGs, enhancing their power density and environmental adaptability to improve energy conversion efficiency is at the core of design objectives. Strategies such as high space utilization design, hybrid generators, and mechanical gain are employed to boost electrical output. Meanwhile, broadband response, multi-directional sensitivity, and hybrid energy harvesting aim to enhance environmental adaptability.

1. Multilayer stacking is indeed an effective method to enhance electrical output within a given spatial volume. Hybrid generators that couple multiple power generation technologies have increased energy capture efficiency through approaches such as broadening the frequency band and energy complementarity. In particular, the application of the magnetic assistance method has significantly enhanced the multifunctionality of the system, which is especially suitable for manufacturing triboelectric-electromagnetic hybrid generators, thereby achieving good mechanical gain effects and increasing the average power density. However, different hybrid mechanisms will lead to differences in the electrical contributions of each unit to the whole. The omnidirectional water wave energy-harvesting unit structure is more practical than the stacking of units with the same structure in all directions. For example, the random rolling of small balls or the adaptive ability of water can enable effective driving under arbitrary water wave conditions.
2. Based on functional structural design, this review delves into the electrical output and power conversion efficiency of devices across various environments. Comparisons of power conversion efficiencies derived from laboratory-based motor-driven tests and simulated underwater environment outputs indicate that most devices achieve efficiencies exceeding 40%. A few studies have also demonstrated good self-powered applications through real-sea testing.
3. In marine energy applications, stable and high electrical output is a key metric for evaluating TENG effectiveness. Meanwhile, device durability and mechanical robustness are essential for long-term use. Many TENGs maintain electrical stability after hundreds of thousands of cycles of operation. Non-contact, rolling, and pendulum-like structures significantly enhance sustainability, enabling the devices to endure harsh marine environments.

### Prospects

**Fig. 17:**
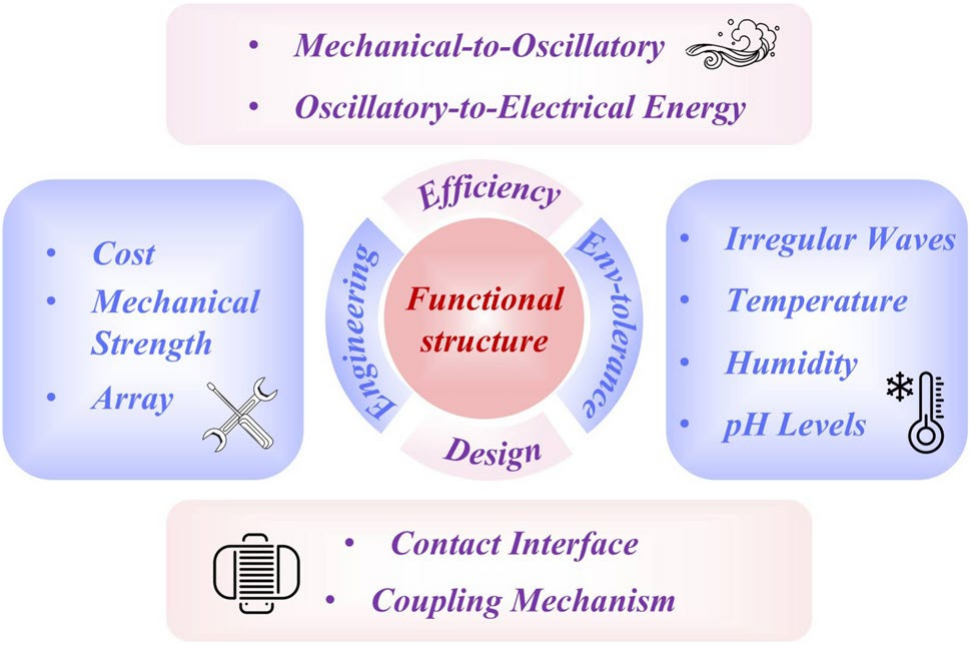
Prospects for developing TENGs to harness water wave energy

Based on the previous discussion about the design of TENGs for harvesting ocean water wave energy, the following presents an outlook for the future development of this field, corresponding to the above-mentioned content. The potential progress in terms of design, efficiency, environmental adaptability, and engineering will be mainly discussed (Fig. 17), and specific solutions and suggestions will be put forward:

**1. Design:** The contact interface, as the crucial part for triboelectrification and charge transfer, determines the efficiency of electricity generation. While the pursuit of "volume density" design represents a crucial avenue for enhancing space utilization. This approach should not merely focus on mechanically packing more power-generating units within a given volume; instead, it requires the development of ingenious electrostatic induction mechanisms.

In addition, the imbalance in power contribution among the units of hybrid generators urgently needs to be addressed. Currently, most hybrid generators are only mechanically combined and it is difficult to achieve a synergistic effect of 1 + 1 > 2. This will lead to system instability and reduced efficiency, affecting the overall power generation performance. Therefore, effective integration of power-generating units and fostering interaction among them are imperative for optimizing hybrid generator performance.

**2. Efficiency**: TENGs ultimately need to operate in a hydrodynamic environment. However, a notable disconnect exists between current research practices and real-world application: the heavy reliance on air-based linear motor simulations for parameter optimization., which cannot accurately reflect the actual operating state of the device in water and thus affects the practicality of the results. As shown in Table 2 of the power conversion ratio, there is still room for improvement in the existing research results. To drive meaningful progress, future research must shift its focus to optimizing TENGs within hydrodynamic environments. This entails understanding the dual-stage energy conversion process—from external wave forces to internal mechanical oscillations, and from oscillations to electrical energy—and addressing inefficiencies across both stages. Currently, most of the literature focuses on the efficiency of the second stage and lacks a comprehensive calculation of the overall energy conversion efficiency from ocean waves to electricity.

**3. Environmental Adaptability**: Despite device stability and mechanical robustness being crucial factors, most existing TENG research focuses on regular water wave conditions. In reality, the ocean's unpredictable wave dynamics, extreme temperature fluctuations (from polar cold to tropical heat), humidity variations, and changes in pH and salinity pose significant challenges. Future studies must assess more systematically how these factors affect TENG performance. Additionally, the long-term impact of corrosion on device lifespan needs urgent attention, as it directly jeopardizes the reliability of ocean-based energy systems.

**4. Engineering**: Although a small amount of literature has involved small-scale array configurations, under real-sea area conditions, there is still a lack of comprehensive and in-depth analysis of large-scale array deployment methods, cost–benefit ratios, and mechanical strength. The cost issue after array configuration is of great significance. However, few studies have comprehensively analyzed the pricing structure of TENGs for blue energy applications. An in-depth cost analysis can not only evaluate the initial investment of the equipment but also optimize maintenance and operation expenses and help determine the most cost-effective design. Conducting such an analysis will contribute to promoting the practical application and commercialization of the technology and ensuring its economic feasibility.
